# Exogenous glycine inhibits root elongation and reduces nitrate-N uptake in pak choi (*Brassica campestris* ssp. *Chinensis* L.)

**DOI:** 10.1371/journal.pone.0204488

**Published:** 2018-09-21

**Authors:** Ruifeng Han, Muhammad Khalid, Jiaxiang Juan, Danfeng Huang

**Affiliations:** 1 Department of Plant Science, School of Agriculture and Biology, Shanghai Jiao Tong University, Shanghai, P. R. China; 2 Key Laboratory of Urban Agriculture (South), Ministry of Agriculture, Shanghai, P. R. China; National Taiwan University, TAIWAN

## Abstract

Nitrogen (N) supply, including NO_3_^-^-N and organic N in the form of amino acids can influence the morphological attributes of plants. For example, amino acids contribute to plant nutrition; however, the effects of exogenous amino acids on NO_3_^-^-N uptake and root morphology have received little attention. In this study, we evaluated the effects of exogenous glycine (Gly) on root growth and NO_3_^-^-N uptake in pak choi (*Brassica campestris* ssp. *Chinensis* L.). Addition of Gly to NO_3_^-^-N agar medium or hydroponic solution significantly decreased pak choi seedling root length; these effects of Gly on root morphology were not attributed to the proportion of N supply derived from Gly. When pak choi seedlings were exposed to mixtures of Gly and NO_3_^-^-N in hydroponic culture, Gly significantly reduced ^15^NO_3_^-^-N uptake but significantly increased the number of root tips per unit root length, root activity and ^15^NO_3_^-^-N uptake rate per unit root length. In addition, ^15^N-Gly was taken up into the plants. In contrast to absorbed NO_3_^-^-N, which was mostly transported to the shoots, a larger proportion of absorbed Gly was retained in the roots. Exogenous Gly enhanced root 1-aminocyclopropane-1-carboxylic acid synthase (ACS) and oxidase (ACO) activities and ethylene production. The ethylene antagonists aminoethoxyvinylglycine (0.5 μM AVG) and silver nitrate (10 μM AgNO_3_) partly reversed Gly-induced inhibition of primary root elongation on agar plates and increased the NO_3_^-^-N uptake rate under hydroponic conditions, indicating exogenous Gly exerts these effects at least partly by enhancing ethylene production in roots. These findings suggest Gly substantially affects root morphology and N uptake and provide new information on the specific responses elicited by organic N sources.

## Introduction

Plant growth and nitrate (NO_3_^-^) accumulation in green leafy vegetables are influenced by the sources of available nitrogen (N) [1]. Consumption of vegetables containing high concentrations of NO_3_^-^ has been related to risks to human health [2]. Plants accumulate NO_3_^-^ when the rate of NO_3_^-^-N uptake through the roots exceeds the rate of NO_3_^-^ assimilation in plant tissues. Given that the roots are the major sites that directly affect NO_3_^-^-N uptake in plants, understanding the effects of different N sources on root morphology is necessary to devise strategies to reduce accumulation of NO_3_^-^ in green leafy vegetables.

N is found in a variety of inorganic and organic forms in soil. NO_3_^-^-N has been traditionally viewed as the main form of inorganic N taken up by plants. However, an increasing number of studies have shown that organic N also contributes to plant N nutrition [3–7]. Amino acids are ubiquitously found in the soil solution and may represent a significant source of N for plants in terrestrial ecosystems. In general, the concentrations of amino acids in soil solutions are very low (< 60 μM) [8, 9]. However, in cropping systems that rely on recycling and decomposition of organic N sources, amino acids may represent a significant N input and important plant-available N pool [10]. Glycine (Gly) is one of the most abundant amino acids and frequently employed as a model amino acid in plant uptake studies because of its low molecular weight [11, 12].

Apart from the direct nutritional effects of N sources, different forms of N function as signals and can control parameters of root morphology, such as root length, lateral root number and root surface area [13–16]. Low concentrations of NO_3_^-^-N stimulate elongation of the lateral roots [17, 18] and lateral root initiation [13], whereas higher NO_3_^-^-N concentrations inhibit root growth [18]. While many researchers have focused on the effects of inorganic N on root growth [13, 17, 18], only a small number of studies have investigated how amino acids regulate root growth [19–20]. For instance, *L*-glutamate (Glu) suppresses primary root length [21–23]. Moreover, while the effects of *L*-Glu on root morphology are relatively well understood; few studies have investigated the response of roots to Gly, a model amino acid in organic N studies [24, 25], and the regulatory interactions between Gly and inorganic N on root growth. Therefore, additional studies on the effects of amino acid N sources on the root morphology of leafy vegetables are necessary.

In addition to root morphology, NO_3_^-^-N uptake can also be affected by the presence of different forms of N and the interactions between these N forms. For example, the presence of exogenous ammonia (NH_4_^+^)-N in nutrient solution reduced the NO_3_^-^-N uptake capacity of crops [26, 27]. Only a small number of studies have investigated the effects of the interactions between amino acids and inorganic N sources on the uptake of NO_3_^-^-N by roots, and most studies on the effects of exogenous Gly on the uptake of NO_3_^-^-N by roots have focused on agricultural crops, such as perennial ryegrass [28] and wheat [9], with only one study on leafy vegetables (Chinese kale) [27].

Root morphology and NO_3_^-^-N uptake can also be influenced by ethylene production [29, 30]. The gaseous hormone ethylene is synthesized from methionine (Met), which can be converted to S-adenosyl *L*-methionine (SAM) by SAM synthetase. SAM is then converted to 1-aminocyclopropane-1-carboxylic acid (ACC) by ACC synthase (ACS), and subsequently to ethylene by ACC oxidase (ACO) [31]. Previous studies have demonstrated ethylene regulates root growth in response to different N sources [30, 32–35]. For example, a high concentration of NO_3_^-^-N activated ethylene production, which inhibited lateral root growth in *Arabidopsis thaliana* [33]. Similarly, Li et al. (2013) reported that NH_4_^+^-N inhibited *Arabidopsis thaliana* lateral root formation by inducing production of ethylene [34]. Domínguez-May et al. (2013) suggested that ethylene also plays a role in the reduced root length induced by Gly in habanero pepper [24]. In addition, NO_3_^-^-N uptake is also regulated by ethylene in the presence of N sources. Zheng et al. (2013) detected rapid production of ethylene under low NO_3_^-^-N conditions, which decreased NO_3_^-^-N uptake [29]. However, only a few studies have evaluated the role of ethylene in exogenous Gly-induced inhibition of root length [21] and reduction of NO_3_^-^-N uptake.

The green leafy vegetable pak choi is commonly cultivated and widely consumed in southern China. However, pak choi tends to accumulate high concentrations of nitrate [36]. Consumption of high levels of nitrate may lead to carcinogenesis and formation of methemoglobin [2]; thus, it would be highly desirable to reduce the accumulation of NO_3_^-^ in leafy vegetable. Therefore, the aim of this study was to investigate the influence of exogenous Gly on root length and NO_3_^-^-N uptake in pak choi. Moreover, the involvement of the ethylene signaling pathway in the changes in root morphology and NO_3_^-^-N uptake observed in response to exogenous Gly application were investigated. We hypothesized that exogenous Gly would reduce root length and decrease NO_3_^-^-N uptake via a mechanism related to increased ethylene production.

## Materials and methods

### Agar plate culture

Seeds of the pak choi cv. ‘Huawang’ were surface sterilized with 70% ethanol for 1 min, rinsed three times in sterile deionized water, infiltrated by soaking in 10% H_2_O_2_ for 5 min, and then extensively rinsed five times with sterile deionized water. The disinfected seeds were placed in 12 cm-diameter sterile Petri dishes containing 50 mL of agar (0.8% *w/v*) culture medium supplemented with 3 mM NaNO_3_. The basic nutrient media (pH 6.0 ± 0.2) was comprised of 1 mM MgSO_4_, 0.5 mM KH_2_PO_4_, 1.25 mM K_2_SO_4_, 2.5 mM CaCl_2_, 0.05 mM EDTA·2Na, 0.05 mM FeSO_4_, 48.5 μM H_3_PO_3_, 10 μM MnSO_4_, 0.8 μM ZnSO_4_, 0.2 μM CuSO_4_, 2.1 μM NaMoO_3_ and 4.8 μM KI. The Petri dishes were half sealed with adhesive tape and vertically oriented in a growth chamber maintained at 25°C (day-time) and 18°C (night-time) under a 16-h/8-h light/dark cycle with a light intensity of 200 μmol·m^-2^·s^-1^ during the day. Seedlings with a 5–6 mm-long primary root were transferred to sterile treatment dishes (four seedlings per dish) filled with 50 mL of solidified treatment medium containing different sources and concentrations of N. The superior segment of the medium in each dish was removed to ensure that the seedling shoots were not in contact with the medium.

### Hydroponic culture

Seeds of pak choi cv. ‘Huawang’ were surface sterilized as described above, then germinated in plastic trays containing autoclaved perlite. The substrate was supplied daily with basic nutrient solution (as described above) and 3 mM NaNO_3_. After 15 d, uniformly sized seedlings were selected and transplanted to a foam board floating on 3 mM NaNO_3_ solution in hydroponic plastic pots and pre-cultivated for 3 d prior to the experiments. The solutions were renewed every 3 d. All plants were grown in a greenhouse at 25°C (day-time)/18°C (night-time) under a 14-h/10-h light/dark cycle with natural sunlight with photosynthetically active radiation in the range of 300–800 μmol·m^-2^·s^-1^ during the day.

### Experiment 1: Effect of Gly concentration on pak choi root development in the presence of NO_3_^-^-N

To determine the effect of Gly on root growth, on agar plates containing treatment medium with either 0.5 or 10 mM NaNO_3_ were supplemented with a range of Gly concentrations (0, 0.5, 1, 2.5, 5, 10 mM). To make the treatment media, sterile-filtered NaNO_3_ or Gly were added separately to autoclaved basic medium that had been cooled to 50–55°C. The amount of sodium (Na) in the medium containing 0.5 mM NaNO_3_ was adjusted to 10 mM by adding Na_2_SO_4_. After 5 d treatment, primary root growth was measured with a ruler and lateral root number was recorded. To investigate whether the effects of Gly on the primary roots were due to altered N supply, seeds were germinated in treatment medium containing 0 mM N, 2.5 mM Gly or NaNO_3_ and a range of NO_3_^-^-N concentrations (0, 0.5 and 10 mM) for 3 d. The amount of sodium (Na) in the medium containing 0 and 0.5 mM NaNO_3_ was adjusted to 10 mM by adding Na_2_SO_4_. The length of primary root elongation was recorded after 24 and 48 h. All agar plate experiments performed twice independently, using five Petri dishes containing four seedlings each for each treatment.

### Experiment 2: Time course of the effects of exogenous Gly on root system parameters and ^15^N uptake

The dynamic changes in root system parameters and NO_3_^-^-N uptake induced by exogenous Gly were investigated in hydroponic culture using nutrient solution containing 10 mM NaNO_3_ or 10 mM NaNO_3_ + 2.5 mM Gly (exogenous Gly). On days 0, 4, 8, 12 and 16 of treatment, 50 seedlings were selected, the roots were washed thoroughly with purified water, patted dry with filter paper and the seedlings were placed individually in 50 mL centrifuge tubes containing pretreatment nutrient solution (10 mM NaNO_3_ or 10 mM NaNO_3_ + 2.5 mM Gly); the tubes were covered with black plastic film to avoid the effects of light on root growth. The seedlings in tubes were preincubated in controlled-environment chambers at 25°C (day-time)/18°C (night-time) under a 16-h/8-h light/dark cycle (200 μmol·m^-2^·s^-1^). After 24 h, the seedlings were transferred to centrifuge tubes filled with Na^15^NO_3_, Na^15^NO_3_ + Gly or ^15^NGly + NaNO_3_ treatment solution. Labelled N was provided as 10 mM 4.95 atom% Na^15^NO_3_ or 2.5 mM 4.95 atom% ^15^NGly (Shanghai Research Institute of Chemical Industry, China). Fifteen pak choi seedlings were subjected to each treatment (3 replicates, 5 seedlings per replicate). In addition, five ‘blank’ seedlings were treated with the same concentration of the unlabeled NaNO_3_ + Gly mixture. To prevent degradation of amino acids by bacteria, ampicillin (10 mg·L^-1^) was added to all solutions [37]. After the 4 h uptake period, the plants were harvested, divided into shoots and roots, and the five seedlings from each replicate were pooled to form single root/shoot samples. The roots were washed with sterile water, then 0.5 mM CaCl_2_, followed by several washes with purified water to remove ^15^N from the root surface. The shoots and roots were dried at 60°C for 72 h, weighed and ground to a fine powder. The N and ^15^N contents of the roots and shoots were analyzed using a Vario EL III IRMS elemental analyzer (Elementar Analysensysteme GmbH, Hanau, Germany).

In addition, after 1, 5, 9, 13 and 17 d of the NaNO_3_ and NaNO_3_ + Gly treatments, the roots were placed in deionized water and scanned using an Epson Perfection V850 Pro scan system (Nagano, Japan). Root morphological parameters were calculated using WinRhizo analysis software (Regent Instruments Inc., Quebec City, Canada). Root activity was determined using the triphenyltetrazolium chloride (TTC) reduction method according to Islam et al. (2007) [38].

### Experiment 3: Effects of exogenous Gly on ethylene production and the activity of ethylene synthesis enzymes

Fifteen-day-old pak choi seedlings (cultured as described in the hydroponic culture section) were pre-cultivated in 3 mM N nutrient solution for 7 d, cultivated in treatment nutrient solution containing 10 mM NaNO_3_ or 10 mM NaNO_3_ + 2.5 mM Gly for 10 or 15 d, then the plant roots were harvested to determine ethylene production and assay the activity of ethylene synthesis enzymes. The roots were washed as described in Experiment 2 to remove NaNO_3_ or Gly on the root surfaces. To minimize wounding effects, the excised roots were weighed and placed in 20 mL gas-tight vials containing 1 mL agar medium (0.7% *w/v*) for 30 min and the vials were sealed with a gas-tight stopper. After incubation at 25°C for 2 h in the dark, 1 mL of headspace gas was collected from the vials and analyzed using a GC-2010 gas chromatograph (Shimadzu, Kyoto, Japan) equipped with a flame ionization detector (FID) to measure the concentration of ethylene. Root 1-aminocyclopropane-1-carboxylic acid synthase (ACS) and oxidase (ACO) activity were measured according to Tian et al. (2009) [33] and Yu et al. (2016) [39].

### Experiment 4: Effects of ethylene biosynthesis inhibitors on root length in the presence of exogenous Gly

The effects of ethylene biosynthesis inhibitors (AVG and AgNO_3_) on root growth in the presence of exogenous Gly were investigated. According to the culture conditions described in Experiment 1, pak choi seedlings were grown for 5 d in treatment medium containing 2.5 mM Gly and 10 mM NaNO_3_ supplemented with 0, 0.5, 1, 2.5, 5 or 10 μM AVG (06665, Sigma-Aldrich) or 0, 5, 10, 20, 30 or 50 μM AgNO_3_. The sterile-filtered Gly, NaNO_3_, AVG or AgNO_3_ solutions were added to autoclaved basic medium cooled to 50–55°C. Primary root length was measured using a ruler after 5 d.

### Experiment 5: Effects of ethylene biosynthesis inhibitors on ^15^N uptake in response to exogenous Gly

The effect of AVG and AgNO_3_ on ^15^NO_3_^-^-N uptake in the presence of exogenous Gly was investigated. Following the culture conditions described for Experiment 2, 15-day-old pak choi seedlings were pre-cultivated for 7 d in nutrient solution containing 3 mM NaNO_3_. The plants were transferred to 50 mL centrifuge tubes containing the following pretreatment solutions: (1) 10 mM NaNO_3_, (2) 10 mM NaNO_3_ plus 0.5 μM AVG, (3) 10 mM NaNO_3_ plus 10 μM AgNO_3_, (4) 10 mM NaNO_3_ + 2.5 mM Gly, (5) 10 mM NaNO_3_ + 2.5 mM Gly plus 0.5 μM AVG, and (6) 10 mM NaNO_3_ + 2.5 mM Gly plus 10 μM AgNO_3_. After 36 h, the plants were transferred to new centrifuge tubes filled with ^15^N-labelled solutions for 4 h for the short-term uptake test. In each treatment, one of the N sources was labelled with ^15^N (4.95 atom%) for the NaNO_3_ + Gly mixtures, either ^15^NO_3_^-^-N (4.95 atom%) or ^15^N-Gly (4.95 atom%) creating a total of 9 treatments (with 3 replicates and 3 plants per replicate). After the 4 h uptake test, sampling and analysis were performed as described in Experiment 2.

### Calculations

NO_3_^-^-N and Gly uptake were calculated as the ^15^N content of treated pak choi seedlings compared to the ^15^N content of “blank” seedlings cultured in unlabeled NO_3_^-^-N and Gly, according to Eq (1) [5, 6].

$${}^{15}N_{uptake}=DW\times N\%\times \frac{A_{s}-A_{c}}{A_{applied}}$$ (1)

Where ^15^*N*_*uptake*_ is the amount of absorbed N source in the roots or shoots, *DW* is the dry weight of the roots or shoots, N% is the N content of the roots or shoots, *A*_*s*_ is the ^15^N atom% in the roots or shoots of the treated seedlings, *A*_*c*_ is the ^15^N atom% in “blank” seedlings provided with unlabeled N sources, and *A*_*applied*_ is the ^15^N atom% used in the experiment (4.95% ^15^NO_3_^-^-N and 4.95% ^15^N-Gly).

The fraction of N derived from each N source in the mixtures of NaNO_3_ + Gly was calculated according to Eq (2).

$$\mathrm{The}\;\mathrm{fraction}\;\mathrm{of}\;N\;\mathrm{derived}\;\mathrm{from}\;N\;\mathrm{source}=\frac{{}^{15}N_{uptake}}{{}^{15}N_{total\;uptake}}\times 100$$ (2)

Where ^15^*N*_*uptake*_ is the amount of absorbed NO_3_^-^-N or Gly in the roots or shoots of plants cultured in a mixture of N sources, and ^15^*N*_*total uptake*_ is the total amount of absorbed NO_3_^-^-N and Gly in the roots or shoots.

^15^N_*uptake rate*_ was calculated according to Eq (3).

$${}^{15}N_{uptake\;rate}=\frac{{}^{15}N_{uptake}}{DW\times 15\times t}$$ (3)

Where ^15^N_*uptake rate*_ is the uptake rate for the N source for whole seedlings, ^15^*N*_*uptake*_ is the total amount of absorbed N source in whole seedlings, *DW* is the dry weight of whole seedlings, 15 is the molecular weight of labelled N, *t* is the duration of the uptake experiment (4 h).

The ^15^N_*uptake rate per unit root length*_ was calculated according to Eq (4).

$${}^{15}N_{uptake\;rate\;per\;unit\;root\;length}=\frac{{}^{15}N_{uptake\;rate}}{TRL}$$ (4)

Where ^15^N_*uptake rate per unit root length*_ is the N uptake rate per unit root length. The ^15^N_*uptake rate*_ is calculated from the Eq (3), and *TRL* is the total root length.

### Statistical analyses

A one-way experimental design was employed. All statistical analyses were performed using SAS software (SAS Institute, Cary, NC, USA). Differences between treatments were analyzed using the Student’s *t*-test (two treatments) or least significant difference test (*LSD*, ≥ three treatments) at *P* < 0.05. Data are presented as the mean ± SE (standard error). Figures were generated using SigmaPlot 10.0 (Systat Software, Inc., Erkrath, Germany).

## Results

### Root morphology and growth

**Fig 1:**
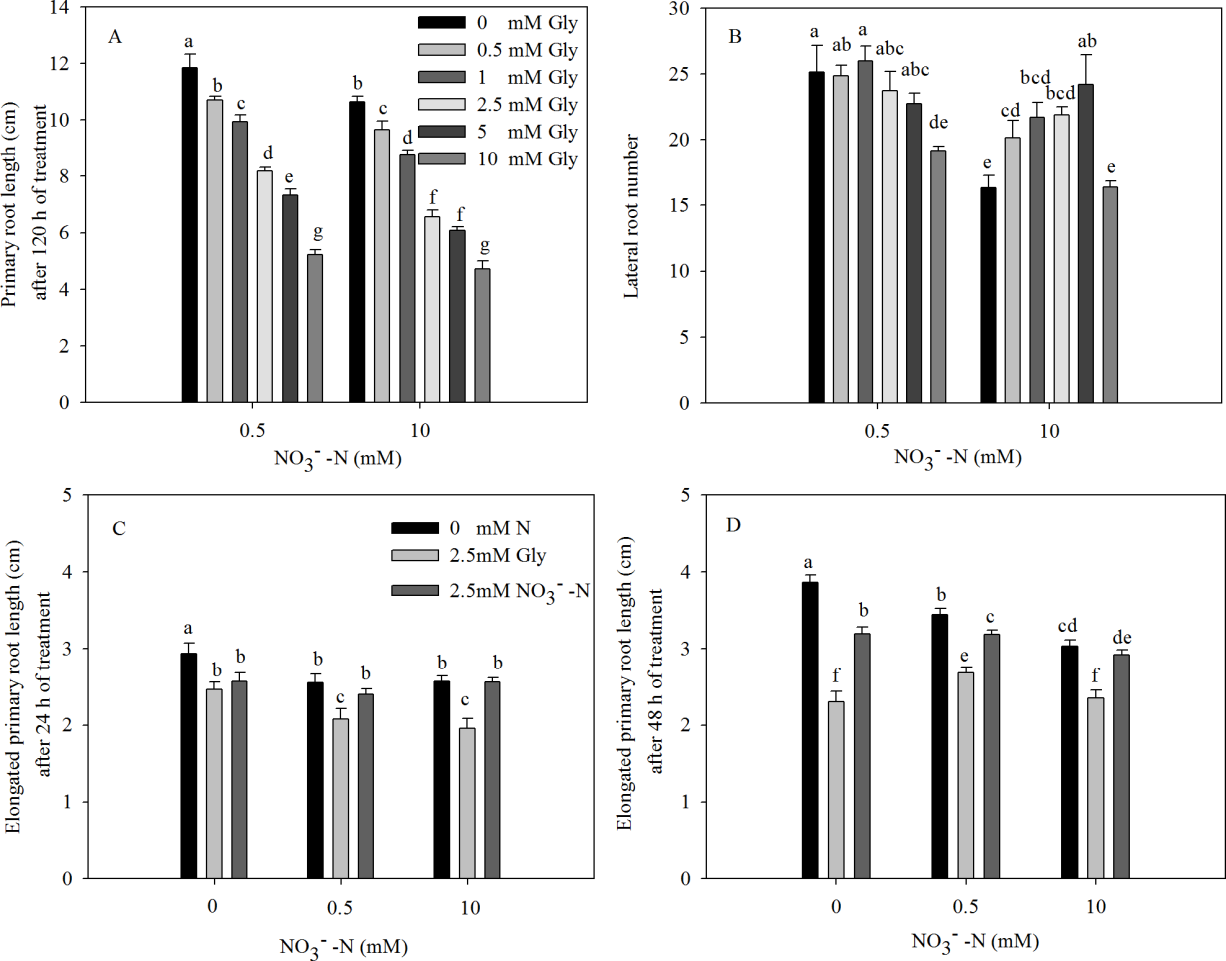
Effect of exogenous Gly and NO_3_^-^-N on the primary root growth. (A) Primary root length and (B) lateral root number of pak choi seedlings grown on axenic agar medium containing 0.5 or 10 mM NO_3_^-^-N and various concentrations of Gly (0–10 mM) for 5 d. (C) Primary root elongation after 24 h treatment and (D) after 48 h treatment under equivalent N concentrations (2.5 mM Gly or NO_3_^-^-N in combination with 0, 0.5 or 10 mM NO_3_^-^-N). The root tips of seedlings grown on agar medium were marked to determine the extent of root elongation every day. Daily differences in primary roots were obtained by measuring two successively dated root lengths. Data are mean ± SE (*n* = 5). Different letters indicate significant differences at *P* < 0.05, *LSD* test.

Pak choi seedlings were co-treated with 0.5 or 10 mM NO_3_^-^-N and varied concentrations of Gly for 5 d on agar plates. In both NO_3_^-^-N treatments, exogenous Gly significantly reduced the primary root length in a concentration-dependent manner compared with NO_3_^-^-N as a single N source (Fig 1A and S1 Fig). The primary root length of pak choi seedlings exposed to 0.5 or 10 mM NO_3_^-^-N supplemented with 2.5 mM Gly was 31.0% and 38.5% lower, respectively, than seedlings exposed to 0.5 and 10 mM NO_3_^-^-N. Thus, 2.5 mM Gly was used in subsequent experiments as this concentration obviously reduced primary root length. The presence of low to medium concentrations of Gly (≤ 5 mM Gly) also significantly increased lateral root number compared to seedlings cultured in the absence of Gly, though the addition of 10 mM Gly did not affect lateral root number in the 10 mM NO_3_^-^-N treatment (Fig 1B). Moreover, the primary root length and lateral root number of 0.5 mM NO_3_^-^-N-treated seedlings were significantly higher than seedlings cultured in 10 mM NO_3_^-^-N (Fig 1A and 1B). Similar results were observed in seedlings supplied with varying concentrations of NO_3_^-^-N (S2 Fig).

To examine whether the response of the roots to Gly was the result of the altered N supply, we assessed the root elongation of seedlings supplied with 2.5 mM NO_3_^-^-N and 2.5 mM Gly (i.e. the same millimolar concentrations of each N source) in the presence of 0, 0.5 or 10 mM NO_3_^-^-N. Compared with equimolar concentrations of NO_3_^-^-N (2.5, 3 or 12.5 mM), the elongated root lengths of seedlings exposed to 2.5 mM Gly were 4.4%, 13.6% and 23.4% shorter during the first 24 h of treatment (Fig 1C) and 27.8%, 15.7% and 19.3% shorter during the second 24 h of treatment than seedlings exposed to 2.5 mM plus 0, 0.5 or 10 mM NO_3_^-^-N (Fig 1D). These results suggest the effects of Gly on root length were not due to a change in the concentration of N available to the roots.

**Fig 2:**
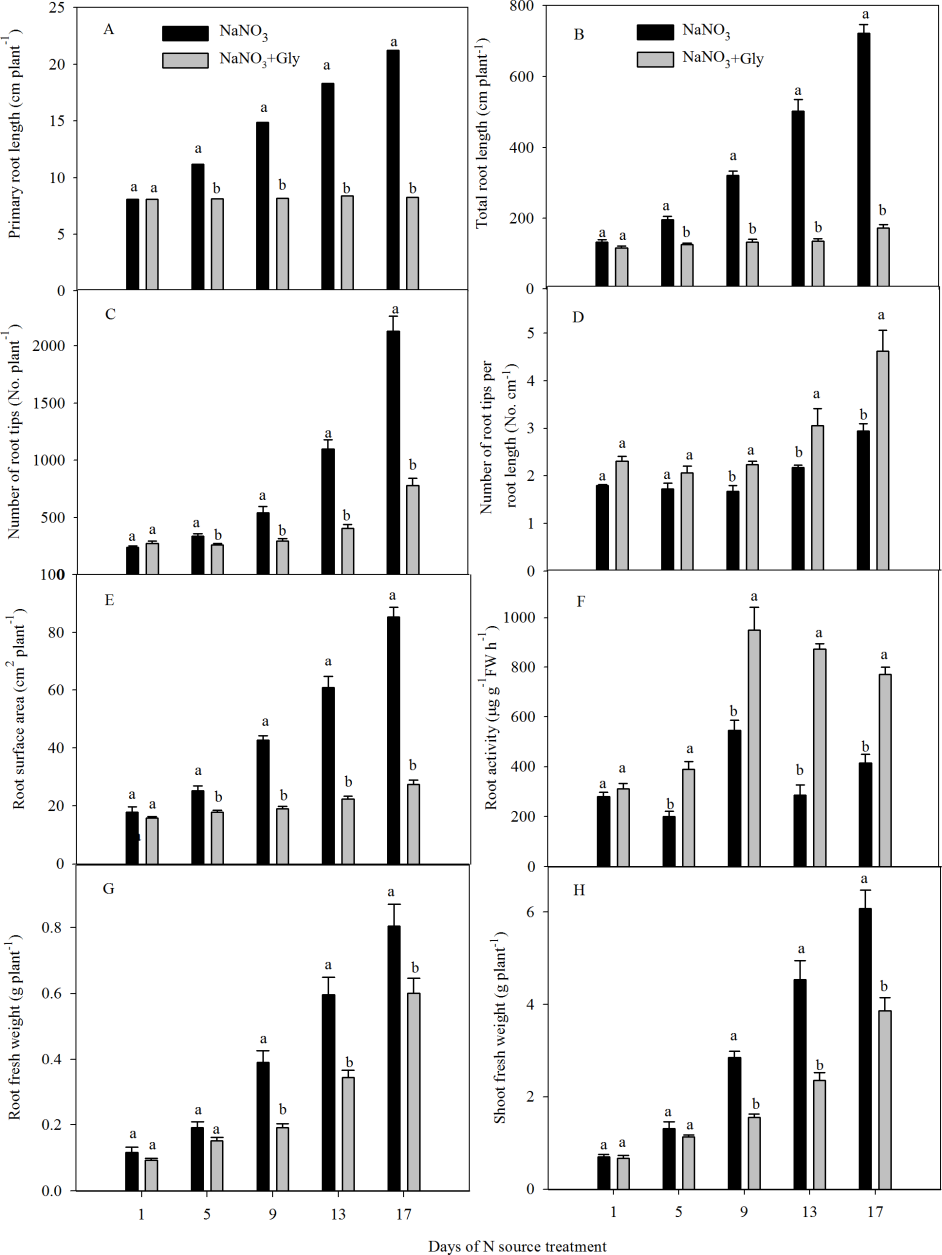
Time course of changes in the root morphological parameters and growth of pak choi seedlings exposed to exogenous Gly. Eighteen-day-old pak choi seedlings were transferred to hydroponic nutrient solution containing 10 mM NaNO_3_ with or without 2.5 mM Gly. (A) Primary root length, (B) total root length, (C) number of root tips, (D) number of roots per root length, (E) root surface area, (F) root activity, (G) root fresh weight and (H) shoot fresh weight of pak choi seedlings at various time points. Data are mean ± SE (*n* = 9). Different letters indicate significant differences between treatments at *P* < 0.05, Student’s *t*-test.

Next, we performed a time course assessment of the changes in root morphological parameters and the fresh weight of pak choi seedlings induced by NaNO_3_ + Gly under hydroponic conditions (Fig 2). Compared with NaNO_3_ alone, NaNO_3_ + Gly decreased the primary root length, total root length, number of root tips and root surface area (Fig 2A, 2B, 2C and 2E and S3 Fig), but increased the number of root tips per unit root length and root activity (Fig 2D and 2F) at 5 d. Moreover, NaNO_3_ + Gly significantly decreased the root fresh weight and shoot fresh weight compared with NaNO_3_ alone after 9 d (Fig 2G and 2H). After 17 d, the primary root length, total root length, number of root tips, root surface area, root and shoot fresh weights of seedlings exposed to NaNO_3_ + Gly were 61.1%, 76.0%, 63.4%, 67.8%, 25.3% and 36.3% lower, and the number of root tips per unit root length and root activity were 57.0% and 95.0% higher than seedlings exposed to NaNO_3_ (Fig 2 and S3 Fig).

### NO_3_^-^-N uptake

**Fig 3:**
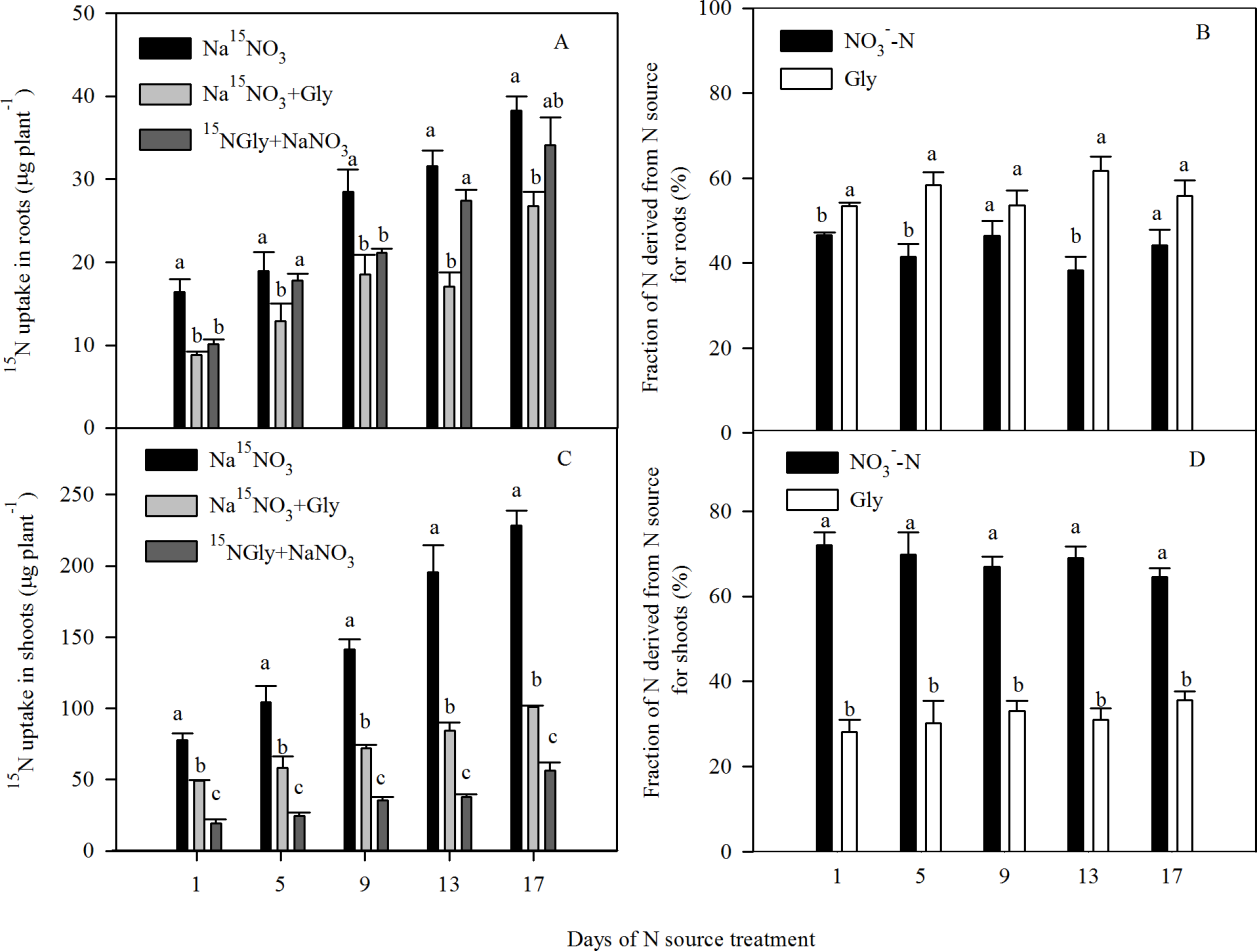
Time course of changes in ^15^N uptake by pak choi seedlings exposed to Na^15^NO_3_, Na^15^NO_3_ + Gly or ^15^NGly + NaNO_3_. (A) Uptake of ^15^N-labelled NO_3_^-^-N and Gly in the roots, (B) fraction of N derived from each N source for the roots, (C) uptake of ^15^N-lablled NO_3_^-^-N and Gly in the shoots, and (D) fraction of N derived from each N source for the shoots. Data are mean ± SE (*n* = 3). Different letters indicate significant differences between treatments at *P* < 0.05, *LSD* test.

Next, we examined the effects of exposure to NaNO_3_ and Gly on the uptake of ^15^N (by using Na^15^NO_3_ and ^15^N-Gly) under hydroponic conditions (Fig 3). Compared to 10 mM NaNO_3_, the addition of Gly significantly decreased ^15^NO_3_^-^-N uptake between days 1 to 17 (Fig 3A and 3C). Moreover, ^15^N-Gly was detectable in seedlings exposed to NaNO_3_ + Gly between days 1 to 17 of treatment. In the NaNO_3_ + Gly treatment, the roots accumulated significantly less ^15^NO_3_^-^-N than ^15^N-Gly, with ^15^NO_3_^-^-N accounting for 38.4–46.8% and ^15^N-Gly accounting for 53.3–61.7% of root N (Fig 3B). In comparison, the shoots accumulated significantly more ^15^NO_3_^-^-N than ^15^N-Gly between days 1 to 17, with ^15^NO_3_^-^-N accounting for 64.3–71.8% of shoot N (Fig 3D). Since shoots have a higher fresh weight than roots, the high fraction of N derived from NO_3_^-^-N in the shoots led to an overall increase in the fraction of whole plant N derived from NO_3_^-^-N. In addition, the ratio of NO_3_^-^-N translocated from the roots to the shoots was significantly higher in seedlings exposed to Na^15^NO_3_ and Na^15^NO_3_ + Gly than seedlings exposed to ^15^NGly + NaNO_3_ (S4 Fig), suggesting N derived from NO_3_^-^ tends to translocate to the shoots, whereas N derived from Gly tends to be retained in the roots.

**Fig 4:**
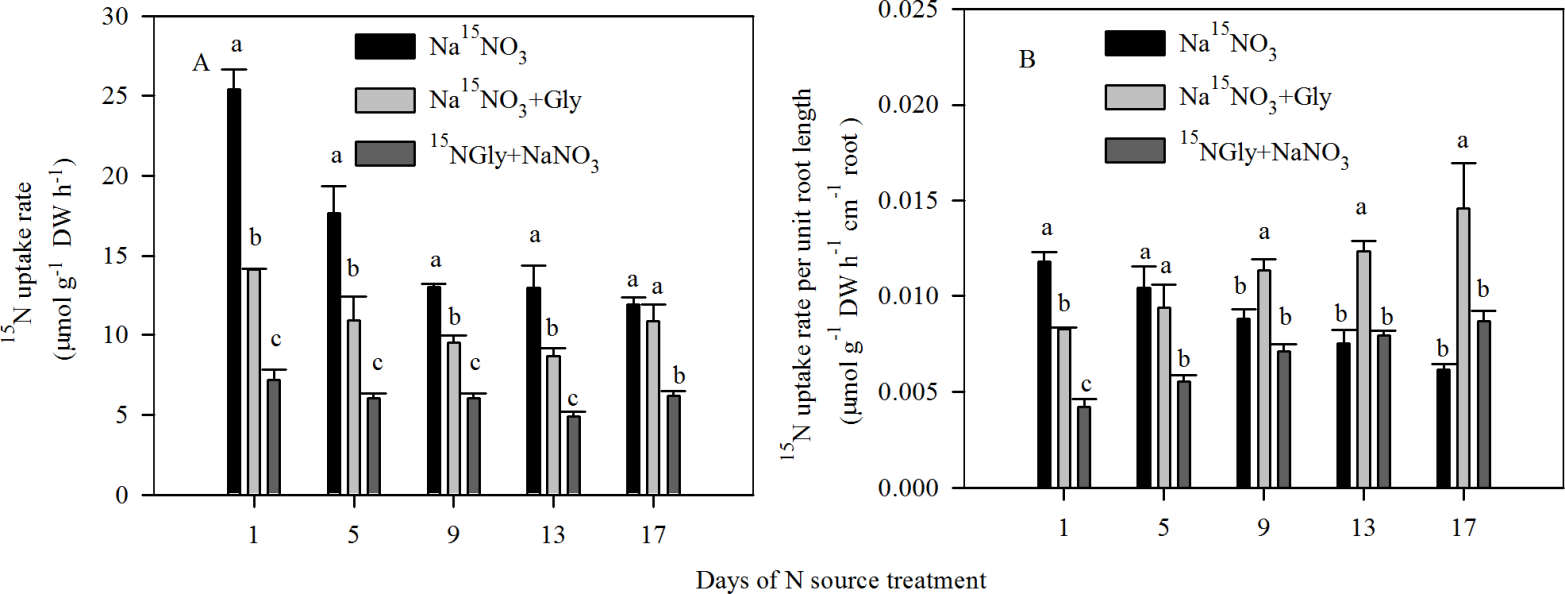
**(A)** ^**15**^**N uptake rate and (B)** ^**15**^**N uptake rate per unit root length over time for pak choi seedlings exposed to Na**^**15**^**NO**_**3**_**, Na**^**15**^**NO**_**3**_ **+ Gly or** ^**15**^**NGly + NaNO**_**3**_. Data are mean ± SE (*n* = 3). Different letters indicate significant differences between treatments at *P* < 0.05, *LSD* test.

The presence of exogenous Gly significantly decreased the ^15^NO_3_^-^-N uptake rate by 8.8–44.6% compared with seedlings exposed to solution containing Na^15^NO_3_ alone between days 1 to 17 of treatment. However, the reduction in the rate of ^15^NO_3_^-^-N uptake was compensated for by a significant increase in the ^15^N-Gly uptake rate, thus, NaNO_3_ + Gly-treated seedlings maintained similar or had even higher ^15^N uptake rates than seedlings exposed to only NaNO_3_ (Fig 4A). In addition, to allow a more complete characterization of the ^15^N uptake in response to the total root length changes that were induced by exogenous Gly, the ^15^N uptake rate per unit of root length was calculated (Fig 4B). After 1 d of treatment, the ^15^N uptake rate per unit of root length was highest in Na^15^NO_3_-treated seedlings, followed by Na^15^NO_3_ + Gly and then ^15^NGly + NaNO_3_-treated seedlings. However, during the course of the 17-day experiment, the ^15^N uptake rate per unit root length gradually decreased in Na^15^NO_3_-treated seedlings, whereas the ^15^N uptake rate per unit root length significantly increased in Na^15^NO_3_ + Gly and ^15^NGly + NaNO_3_-treated seedlings over time (Fig 4B). At 17 d, the ^15^NO_3_^-^-N uptake rate per unit of root length was 1.4-fold higher in Na^15^NO_3_ + Gly-treated plants than Na^15^NO_3_-treated plants (Fig 4B).

### Ethylene and activities of ethylene synthesis enzymes in the root

In seedlings exposed to NaNO_3_ + Gly, uptake of Gly resulted in significant accumulation of free amino acids in the roots, including a 31.43% increase in the levels of the ethylene precursor methionine (Met) compared with the roots of NaNO_3_-treated seedlings (S1 Table). This finding prompted us to examine whether exposure to NaNO_3_ + Gly altered ethylene production. The roots of seedlings grown in NaNO_3_ + Gly produced significantly higher levels of ethylene than seedlings grown in NaNO_3_ (Table 1). Moreover, the roots of seedlings exposed to NaNO_3_ + Gly had higher ACS and ACO activities compared with seedlings treated with NaNO_3_ after 10 and 15 d treatment (Table 1).

**Table 1:** Effects of exogenous Gly on ethylene production and ACS and ACO activity in the roots of pak choi seedlings.

Treatment	10 days			15 days		
	Ethylene (nl C_2_H_4_·g^-1^ FW·h^-1^)	ACS activity (nl C_2_H_4_·g^-1^ FW·h^-1^)	ACO activity (nl C_2_H_4_·g^-1^ FW·h^-1^)	Ethylene (nl C_2_H_4_·g^-1^ FW·h^-1^)	ACS activity (nl C_2_H_4_·g^-1^ FW·h^-1^)	ACO activity (nl C_2_H_4_·g^-1^ FW·h^-1^)
NaNO_3_	1.70±0.06b	0.80±0.04b	3.23±0.21b	n.d.	0.93±0.1b	1.08±0.07b
NaNO_3_+Gly	2.75±0.26a	1.46±0.13a	9.28±0.36a	n.d.	1.73±0.16a	7.58±0.46a

n.d., not determined. Data are mean ± SE (*n* = 4). Different letters indicate significant differences within columns at *P* < 0.05, Student’s *t*-test.

### The role of ethylene in exogenous Gly-induced changes in root elongation and ^15^NO_3_^-^-N uptake

**Fig 5:**
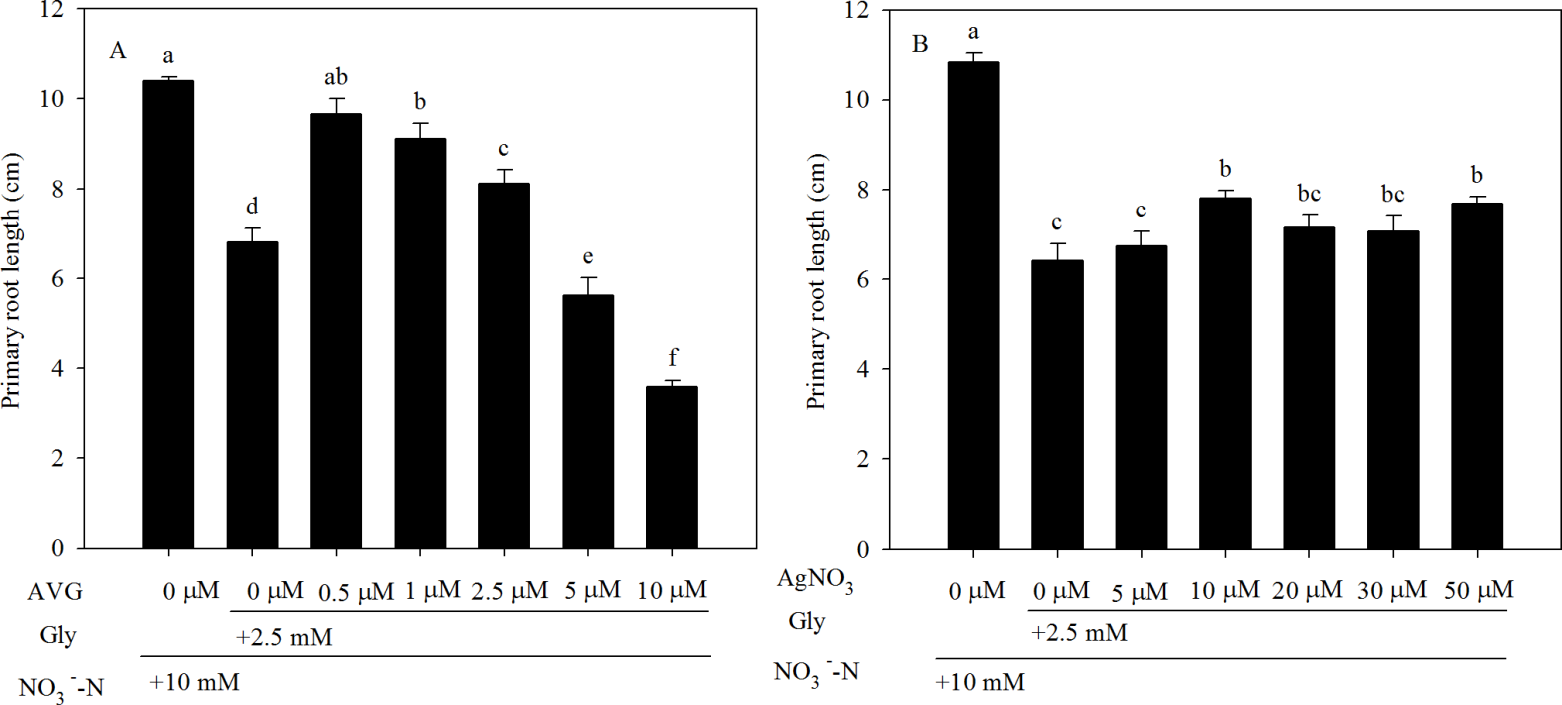
**Effect of the ethylene synthesis inhibitors (A) AVG and (B) AgNO_3_ on the inhibition of primary root length induced by exogenous Gly**. Pak choi seedlings were grown on axenic agar medium containing 10 mM NO_3_^-^-N with 2.5 mM Gly and various concentrations of AVG (0–10 μM) or AgNO_3_ (0–50 μM) for 5 d. Data are mean ± SE (*n* = 5). Different letters indicate significant differences at *P* < 0.05, *LSD* test.

To determine whether the inhibitory effect of exogenous Gly on root elongation were due to altered ethylene production, we investigated the effects of the ethylene inhibitor AVG and ethylene perception blocker AgNO_3_ on the primary root length of pak choi seedlings grown in NaNO_3_ + Gly agar medium. Exposure to NaNO_3_ + Gly significantly reduced primary root length compared with NO_3_^-^-N. However, AVG (≤ 2.5 μM) markedly reversed the inhibition of primary root length induced by NaNO_3_ + Gly (Fig 5A, S5A Fig); 0.5 μM AVG had the most significant effect. Moreover, 10 μM AgNO_3_ led to a partial recovery of primary root length in seedlings exposed to NaNO_3_ + Gly (Fig 5B, S5B Fig). Additionally, compared to treatment with NaNO_3_ + Gly, the presence of 0.5 μM AVG or 10 μM AgNO_3_ significantly increased the primary root length (by 41.9% and 21.7%, respectively).

**Fig 6:**
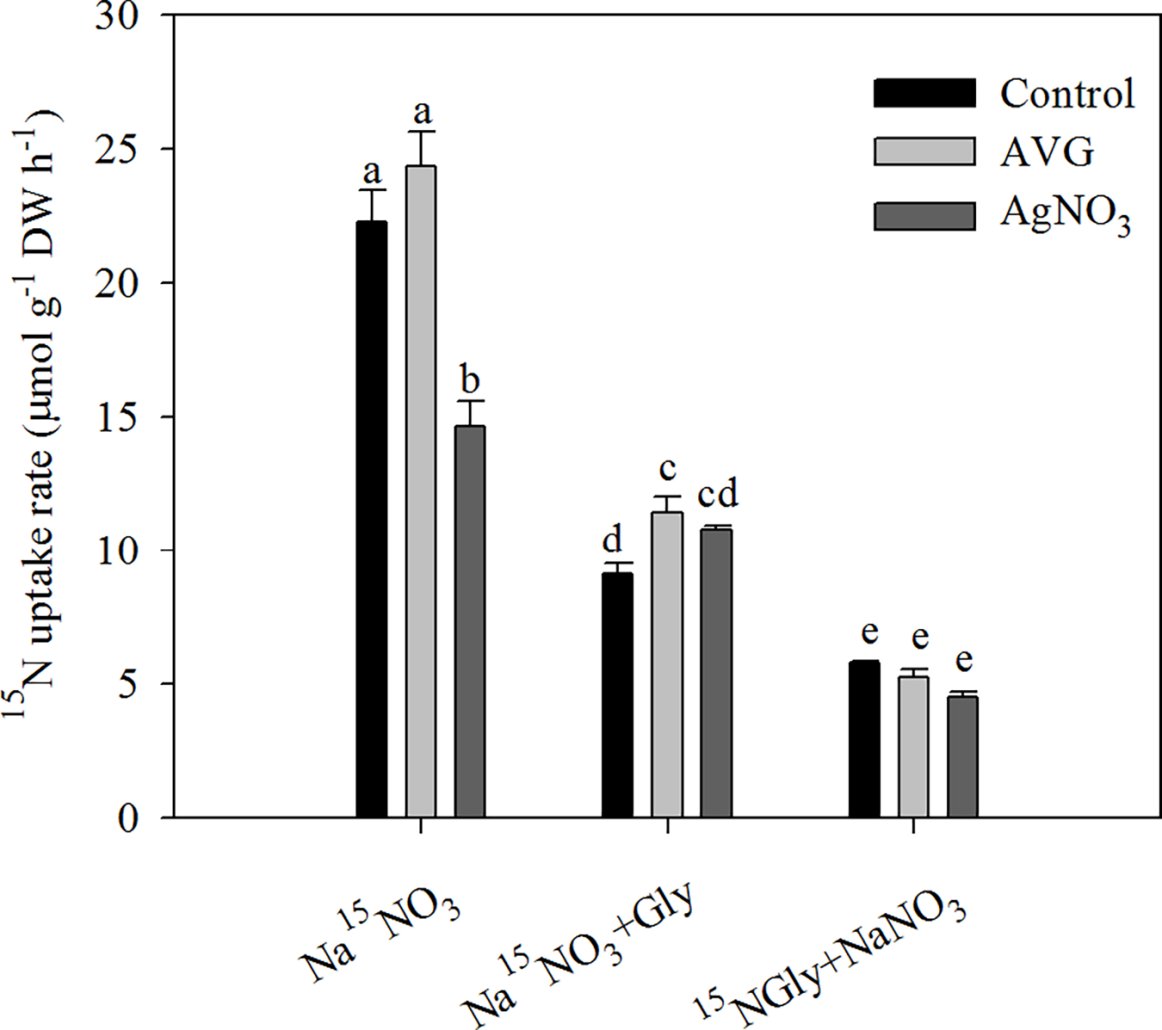
Effect of AVG or AgNO_3_ and exogenous Gly on the ^15^N uptake rate of pak choi seedlings. Twenty two-day-old pak choi seedlings were exposed to nutrient solution containing 10 mM NO_**3**_^-^-N with or without 2.5 mM Gly in the presence or absence of 0.5 μM AVG or 10 μM AgNO_3_ for 4 h. Data are mean ± SE (*n* = 3). Different letters indicate significant differences at *P* < 0.05, *LSD* test.

**Fig 7:**
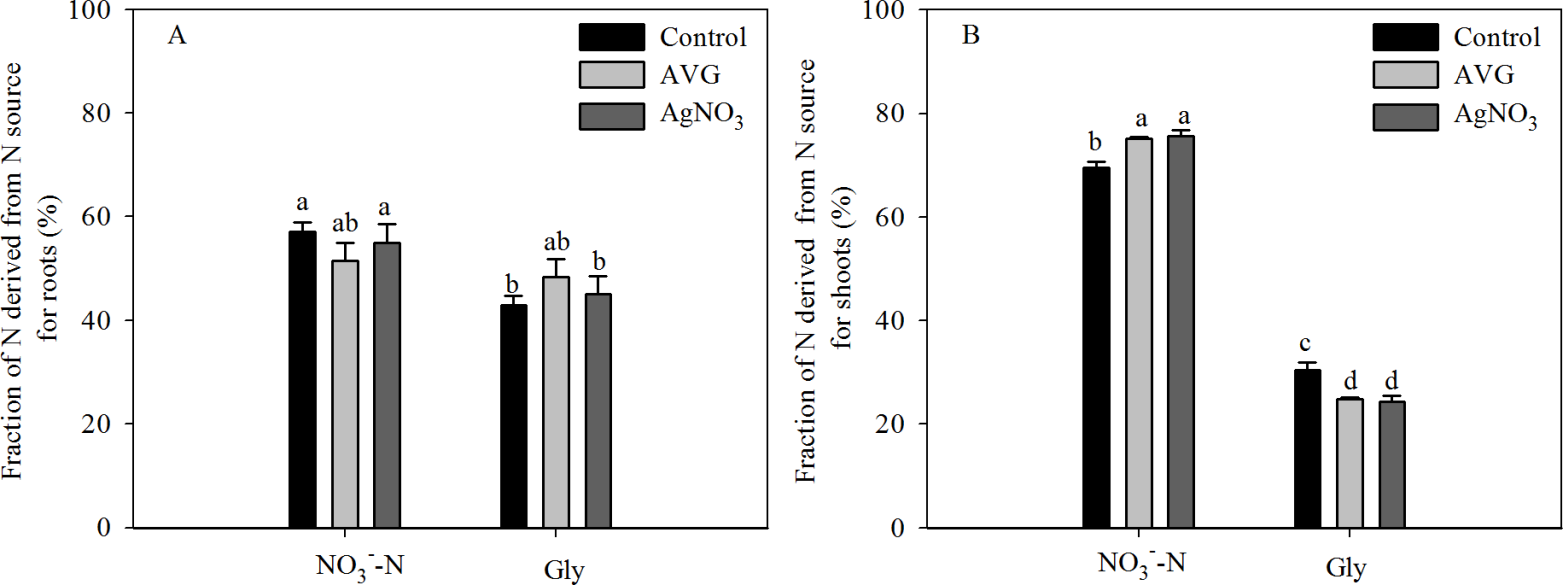
**Effect of AVG or AgNO**_**3**_ **on the fraction of N derived from each N source (NO**_**3**_^-^-**N or Gly) in the (A) roots and (B) shoots.** Twenty two-day-old pak choi seedlings were exposed to nutrient solution containing 10 mM NO_**3**_^-^-N with 2.5 mM Gly in the presence or absence of 0.5 μM AVG or 10 μM AgNO_3_ for 4 h. Data are mean ± SE (*n* = 3). Different letters indicate significant differences at *P* < 0.05, *LSD* test.

To assess the effect of ethylene in the reduction in the ^15^NO_3_^-^-N uptake rate induced by exogenous Gly, we investigated the effects of 0.5 μM AVG and 10 μM AgNO_3_ on the ^15^N uptake rate in NaNO_3_ + Gly-treated seedlings under hydroponic conditions (Fig 6). Compared with Na^15^NO_3_, addition of 2.5 mM Gly (Na^15^NO_3_ + Gly) or 2.5 mM NaNO_3_ (Na^15^NO_3_ + NaNO_3_) significantly decreased the ^15^NO_3_^-^-N uptake rate; however, there was an approximately 1.2-fold difference in the ^15^NO_3_^-^-N uptake rate between the Na^15^NO_3_ + Gly and Na^15^NO_3_ + NaNO_3_ treatments (Fig 6 and S6 Fig). In NaNO_3_-treated plants, the ^15^NO_3_^-^-N uptake rate was slightly increased by 0.5 μM AVG, but significantly reduced by 10 μM AgNO_3_; in NaNO_3_ + Gly-treated plants, the ^15^NO_3_^-^-N uptake rate was significantly increased and the ^15^N-Gly uptake rate was slightly decreased by 0.5 μM AVG or 10 μM AgNO_3_. Moreover, significant differences in the fraction of N derived from the NO_3_^-^-N source were observed in the shoots between treatments, but not in the roots (Fig 7). In the NaNO_3_ + Gly-treated seedlings, 0.5 μM AVG or 10 μM AgNO_3_ significantly increased the fraction of shoot N derived from the NO_3_^-^-N source (Fig 7B).

## Discussion

Studies typically quantify the effect of exogenous Gly on the NO_3_^-^ content and other physiological responses of leafy vegetables grown in hydroponic culture [4, 5, 27, 40, 41]. Furthermore, hydroponic conditions has been employed to study root morphology and NO_3_^-^-N uptake in Chinese cabbage [42], cucumber [43] and tomato [44], as well as the role of ethylene in the modulation of root length in wheat [40]. Therefore, we used a hydroponic system to assess the effect of exogenous Gly on the root morphology and NO_3_^-^-N uptake and verify the role of ethylene in these processes in pak choi. However, to more easily observe the changes in root length (i.e. without large numbers of branching roots) after 24 or 120 h treatment, we used an agar plate system to investigate the effect of exogenous Gly and AVG/AgNO_3_ on primary root elongation. We confirmed that Gly induced similar reductions in primary root length of pak choi in the agar plate system and hydroponic system (Figs 1 and 2). In hydroponic culture, exogenous Gly suppressed root length and reduced NO_3_^-^-N uptake (Figs 3 and 4). Plant root morphology is an important variable required to ensure adequate access to NO_3_^-^-N, which in turn influences accumulation of NO_3_^-^ [42]. The inhibition of root length and reduction in NO_3_^-^-N uptake induced by Gly in this study may explain how exogenous amino acids decrease the NO_3_^-^ concentration in plants (S7 Fig). Additionally, exogenous Gly also enhanced production of ethylene in the roots of hydroponically grown pak choi (Table 1), and ethylene was at least partly involved in the changes in root development and NO_3_^-^-N uptake induced by exogenous Gly.

### Exogenous Gly inhibits root elongation

In both the agar plate and hydroponic systems, exogenous Gly significantly reduced primary root length (Fig 1A, 1C and 1D, Fig 2A and S1 Fig). This is similar to the effects of single amino acids, such as *L*-Glu and Gly [21, 24, 45] and mixtures of N sources supplied with NO_3_^-^-N and *L*-Glu [19, 20] on plants grown on agar plates. Only Walch-Liu, Forde (2008) and Leblanc et al. (2013) have investigated the effect of Glu on root development in the presence of both NO_3_^-^-N and Glu; the mixtures used in those studies more closely resemble soil conditions. Moreover, NO_3_^-^-N partially antagonized that ability of *L*-Glu to inhibit root length in *Arabidopsis thaliana* [20]. However, unlike Walch-Liu and Forde (2008), we found low and high concentrations of NO_3_^-^-N increased and inhibited, respectively, the primary root length of agar plate-grown pak choi in the presence of Gly (Fig 1A), indicating that NO_3_^-^-N and Gly may exert partially antagonistic (at low NO_3_^-^-N concentrations) or synergistic (at high NO_3_^-^-N concentrations) effects on the growth of the primary roots. Bonner et al. (1996) reported that the inhibitory effect of Gly was probably related to the Glutamine-reversible phenomenon of ‘general amino acid inhibition’ [46]. In this study, the inhibitory effect was attributable to the impact that the exogenous Gly may have on plant metabolism. Low concentrations of NO_3_^-^-N can stimulate the primary root growth directly [20]. Walch-Liu and Forde (2008) suggested that direct stimulation of primary root growth by NO_3_^-^-N might be a manifestation of the same phenomenon as NO_3_^-^-N antagonism of the inhibitory effect of amino acid on primary root growth. In this context, primary root growth may be negatively regulated by the endogenous amino acids pool [20], and low concentrations of NO_3_^-^-N would promote the primary root growth by alleviating this effect. Nevertheless, the high concentrations of NO_3_^-^-N inhibited the primary root growth independently of the presence of exogenous Gly (S2 Fig). It has been suggested that the same NO_3_^-^-N signaling pathway that operates in the lateral roots may also regulate primary root growth [20]. The development of lateral roots has been suggested to be inhibited by the accumulation of N metabolites in the roots [47]. The inhibition of primary root growth at high NO_3_^-^-N concentrations would be negatively regulated by the accumulation of N metabolites. Therefore, we proposed that high concentrations of NO_3_^-^-N further aggravated the inhibition of primary root growth induced by exogenous Gly. In addition, these findings showed that under agar plate growth conditions, exogenous Gly affected root morphology in a manner distinct to NaNO_3_ (S2 Fig). When added at the same millimolar N concentrations, exogenous Gly inhibited primary root growth more severely than NaNO_3_ (Fig 1C and 1D), demonstrating that the effect of Gly on primary root length are not directly attributed to the nutritional effects of N availability.

Amino acids such as Gly modulate root development when present in the growth medium at concentrations higher than 0.5 mM. However, all experiments in this study were completed under sterile conditions, thus eliminating microorganisms, which are considered to be more competitive for organic N than plants. Studies using sterile cultures do not always reflect the actual soil environment, including the forms and concentrations of N and turnover rates of organic N. While the concentrations of amino acids in soil solutions are low [28], concentrations in excess of the levels needed to affect root morphology are likely to occur within soils that absorb substantial quantities of amino acids [48] and decomposing organic matter, which contain millimolar levels of amino acids [10]. Nevertheless, knowledge of whether such high concentrations of amino acids exert unrecognized or negligible effects on root morphology in the field is still lacking. The findings presented in this study help to further understand the effect of organic N on plant root morphology in the presence of both NO_3_^-^-N and organic N, which more closely reflects field conditions. Furthermore, the results of the present study contribute to the existing knowledge that has been generated using inorganic N or single organic N source test systems.

### Exogenous Gly reduces NO_3_^-^-N uptake but gradually increases NO_3_^-^-N uptake rate per unit of root length

In addition to the inhibition of primary root length, exogenous Gly significantly reduced NO_3_^-^-N uptake (rate) from day 1 to 17 of treatment (Figs 3, 4A and 6) under hydroponic conditions. These results are consistent with earlier studies showing that Glu reduced the uptake of NO_3_^-^-N by plants grown on agar plates [19] and Gly reduced the uptake of NO_3_^-^-N under hydroponic conditions [9]. The effects of amino acids may be due to accumulation of N metabolites such as assimilated amino acids. NO_3_^-^-N uptake has been shown to be inhibited by an increase in the concentrations of the downstream products of N sources [49].

In contrast to the reductions in root length and NO_3_^-^-N uptake, after 9 d of treatment, the NO_3_^-^-N uptake rate per unit root length exhibited the opposite trend to that of NO_3_^-^-N uptake (rate), with NaNO_3_ + Gly-treated seedlings having smaller roots but a higher NO_3_^-^-N uptake rate per unit root length, implying a compensatory mechanism related to NO_3_^-^-N uptake by the roots (Fig 4B). Although this compensatory effect was observed, it was unable to restore shoot and root growth and ^15^N uptake to the normal levels (Fig 3A and 3D). In addition, given that the root structure induced by N sources need long-term compensatory mechanisms [50] and that root activity reflects the capacity of the plant root system for nutrient uptake [51], the increased ^15^NO_3_^-^-N uptake rate per unit root length may be positively correlated with the increased number of root tips per unit root length (Fig 2D) and higher root activity (Fig 2F). These results do not exclude the possibility that the increases in the number of root tips per unit root length, root activity and ^15^NO_3_^-^-N uptake rate per unit root length could reflect an important adaptive response to mixtures of Gly and NO_3_^-^-N.

### Role of Gly in pak choi N nutrition

Plants are able to take up amino acids as a source of N [3, 52], and we confirmed Gly was absorbed by pak choi seedlings (Figs 3, 4 and 6). For pak choi grown in mixtures of Gly and NO_3_^-^-N, ^15^N-Gly uptake accounted for 28.2–35.7% of shoot N and 53.3–61.7% of root N (Fig 3B and 3D). However, these fractions may be overestimated, as the plants were grown under sterile conditions in this study. Accurate methods of determining the quantitative contribution of amino acid N in the natural environment have not yet been devised, and are likely to be affected by competition with microorganisms; hence, the actual contribution of Gly to plant nutrition cannot be determined. In addition, Cambui et al. (2011) suggested that a significant share of absorbed amino acids resided, and was incorporated, at the site of primary assimilation [53]. Thus, we found N derived from Gly was more abundant in the roots than shoots (Fig 3 and S4 Fig), indicating that absorbed Gly may be preferentially metabolized in the roots, and slowly transported to the shoots [6].

Moreover, our data showed that the inhibition of NO_3_^-^-N uptake induced by Gly in pak choi seedlings under hydroponic conditions was compensated for by an increase in ^15^N-Gly uptake in order to maintain a similar total N uptake rate (Figs 4 and 6). However, despite N being taken up at a similar rate, the root length and shoot growth of exogenous Gly-treated seedlings were significantly impaired by Gly (Fig 2 and S3 Fig). Phytohormones have been demonstrated to be involved in growth inhibition [24, 54]. In this study, the growth retardation induced by Gly may be related to altered phytohormone levels.

### Ethylene may be involved in the metabolism of absorbed Gly and participate in the regulation of root development and NO_3_^-^-N uptake induced by exogenous Gly

Ethylene can be induced in response to different N sources [34, 55–58]. Our results suggest that exogenous Gly treatment enhanced ethylene production in the roots of pak choi (Table 1). Enhancement of ethylene production may be due to increased synthesis of amino acids (S1 Table) as explained by Kaack and Pedersen (2014) [59]. Moreover, Gly in the roots is incorporated into serine (Ser) and then converted to other amino acids via transamination [6]. The coupled increases in the Gly and Ser contents observed in the root tissues of hydroponically grown pak choi co-treated with NaNO_3_ + Gly (S1 Table) indicate intact Gly can be absorbed by the plants [7, 52]. A previous study suggested absorbed Gly would likely be metabolized to Met [25], the precursor of ethylene [60]. Indeed, we observed an increase in the content of Met (S1 Table). Additionally, the activities of ACS and ACO, two key enzymes responsible for ethylene synthesis in plants, were significantly higher in plants treated with NaNO_3_ + Gly than plants treated with only inorganic N sources (Table 1). In the ethylene synthesis pathway, SAM is the intermediate product [60] and an important methyl donor, while SAM synthase is a vital enzyme that directs the flux of SAM and directly participates in the metabolism of Gly [4]. In a previous study, we reported that SAM synthase was upregulated by Gly [4]. Thereby, these results indicate ethylene may be involved in the pathways by which absorbed Gly is metabolized in plants. These findings provide further evidence that nutritional and hormonal cues collectively regulate the growth of plants.

Ethylene can inhibit root growth and regulate NO_3_^-^-N uptake [29, 61]. In the present study, the inhibition of root growth and NO_3_^-^-N uptake observed in response to mixtures of Gly and NO_3_^-^-N were related to increased ethylene production (Table 1). The ethylene inhibitors AVG and AgNO_3_ attenuated the effects of ethylene under both agar plate and hydroponic conditions [39, 62]. One important finding of this study is that application of an ethylene biosynthesis inhibitor (0.5 or 1 μM AVG) or perception blocker (10 μM AgNO_3_) to NaNO_3_ + Gly-treated pak choi markedly alleviated the inhibition of primary root length under agar plate conditions (Fig 5 and S5 Fig). These findings are in agreement with Domínguez-May et al. (2013), who suggested that ethylene played a regulatory role in the inhibitory effects of Gly in habanero pepper [24]. Moreover, we also showed that exogenous application of 0.5 μM AVG and 10 μM AgNO_3_ could markedly increase the ^15^NO_3_^-^-N uptake rate in hydroponically grown pak choi seedlings in response to NaNO_3_ + Gly (Figs 6 and 7). Our results are consistent with Zheng et al. (2013), who reported ethylene negatively affected the ^15^NO_3_^-^-N uptake rate [29]. However, previous studies adopted both pharmacological and transgenic approaches to investigate the roles of ethylene in root development [33] and NO_3_^-^-N uptake [29]. Therefore, application of transgenic lines will be required in future studies to further elucidate the mechanisms by which Gly induces inhibitory effects on root morphology and reduces NO_3_^-^-N uptake.

## Conclusion

The results presented here clearly show that the root morphological responses of pak choi to Gly and nitrate-N were different to those of seedlings exposed to a single nitrate-N source. Compared to the nitrate-N supply, addition of Gly inhibited the root elongation of pak choi seedlings, and this inhibitory effect was attributed to the specific N forms, rather than the total N concentration. When treated with mixed N sources, pak choi seedlings took up N in the form of nitrate-N and Gly. Furthermore, nitrate-N uptake was reduced by application of Gly. The inhibition of root growth and reduction in nitrate-N uptake induced by Gly was probably mediated by phytohormones, as the roots of pak choi supplied with Gly and nitrate-N showed enhanced production of ethylene. Further investigation also confirmed that the inhibition of root growth and reduction in nitrate-N uptake observed in the presence of Gly were partly related to an increase in ethylene levels. However, the mechanism underlying this phenomenon is not yet fully understood and will remain the focus of further investigations.

## Supporting information

S1 Fig Root growth of pak choi seedlings grown on axenic agar medium containing (A) 0.5 mM or (B) 10 mM NO_3_^-^-N and a range of concentrations of Gly for 5 d.
(TIF)

S2 Fig **Effect of NO_3_^-^-N supply on the (A) primary root length and (B) lateral root number of pak choi seedlings cultured for 4 d on agar plates.** Data are mean ± SE (*n* = 5). Different letters indicate significant differences at *P* < 0.05, *LSD* test.
(TIF)

S3 Fig Phenotypes of pak choi seedlings exposed to NaNO_3_ or NaNO_3_ + Gly. Eighteen-day-old pak choi seedlings were transferred to nutrient solution containing 10 mM NaNO_3_ with or without 2.5 mM Gly and harvested after 17 d.
(TIF)

S4 Fig Time course of percentages of each form of ^15^N translocated from roots to shoots by pak choi seedlings exposed to Na^15^NO_3_, Na^15^NO_3_ + Gly or ^15^NGly + NaNO_3_. Data are mean ± SE (*n* = 3). Different letters indicate significant differences between treatments at *P* < 0.05, *LSD* test.
(TIF)

S5 Fig **Effect of (A) 1 μM AVG and (B) 10 μM AgNO**_**3**_ **in the presence of 10 mM NaNO**_**3**_ **with or without 2.5 mM Gly on the primary root length elongation of pak choi seedlings on agar plates in the first 24 h.** Data are mean ± SE (*n* = 5). Different letters indicate significant differences at *P* < 0.05, *LSD* test.
(TIF)

S6 Fig Effect of exogenous Gly on the ^15^NO_3_^-^-N uptake rate of pak choi seedlings. Twenty two-day-old pak choi seedlings were exposed to nutrient solution containing 10 mM Na^15^NO_3_, 10 mM Na^15^NO_3_ + 2.5 mM NO_**3**_^-^-N, or 10 mM Na^15^NO_**3**_ + 2.5 mM Gly for 4 h. Data are mean ± SE (*n* = 3). Different letters indicate significant differences at *P* < 0.05, *LSD* test.
(TIF)

S7 Fig Effect of exogenous Gly on the nitrate contents of the shoots and roots of pak choi seedlings. Eighteen-day-old pak choi seedlings were transferred to nutrient solution containing 10 mM NO_**3**_^-^-N with or without 2.5 mM Gly for 5 d. Data are mean ± SE (*n* = 4). Different letters indicate significant differences between treatments at *P* < 0.05, Student’s *t*-test.
(TIF)

S1 Table Effects of exogenous Gly on the concentrations of amino acids (μg·g^-1^ FW) in the roots of pak choi seedlings after 5 d treatment under hydroponic culture conditions. (DOT)

## References

[1] Chen GL, Gao XR, Zhang XB. Effect of partial replacement of nitrate by amino acid and urea on nitrate content of non-heading Chinese cabbage and lettuce in hydroponic condition. Journal of Integrative Agriculture. 2002; 1(4): 444–449.

[2] Mobini M, Khoshgoftarmanesh AH, Ghasemi S. The effect of partial replacement of nitrate with arginine, histidine, and a mixture of amino acids extracted from blood powder on yield and nitrate accumulation in onion bulb. Scientia Horticulturae. 2014; 176: 232–237. 10.1016/j.scienta.2014.07.014 PMID: 000342258400030.

[3] Wang XL, Han RF, Tang DM, Huang DF. Comparison of glycine uptake by pak choi in organic and conventional soil under different glycine concentrations: A pot study. Journal of Plant Nutrition and Soil Science. 2015; 178(5): 768–775. 10.1002/jpln.201400587 PMID: 000362575900009.

[4] Wang XL, Tang DM, Huang DF. Proteomic analysis of pak choi leaves and roots under glycine-nitrogen conditions. Plant Physiology and Biochemistry. 2014; 75: 96–104. 10.1016/j.plaphy.2013.12.012 PMID: 000331496000011. 24429133

[5] Xiaochuang C, Lianghuan W, Ling Y, Xiaoyan L, Yuanhong Z, Qianyu J. Uptake and uptake kinetics of nitrate, ammonium and glycine by pak choi seedlings (*Brassica Campestris* L. ssp. *Chinensis* L. *Makino*). Scientia Horticulturae. 2015; 186: 247–253. 10.1016/j.scienta.2015.02.010 PMID: 000353730200032.

[6] Ma Q, Cao X, Wu L, Mi W, Feng Y. Light intensity affects the uptake and metabolism of glycine by pak choi (*Brassica chinensis* L.). Scientific Reports. 2016; 6: 21200. doi: 2120010.1038/srep21200. PMID: 000370382700001. 10.1038/srep21200 26882864 PMC4756379

[7] Liu X, Yang X, Wang L, Duan Q, Huang D. Comparative analysis of metabolites profile in spinach (*Spinacia oleracea* L.) affected by different concentrations of Gly and nitrate. Scientia Horticulturae. 2016; 204: 8–15. 10.1016/j.scienta.2016.02.037.

[8] Jones DL, Owen AG, Farrar JF. Simple method to enable the high resolution determination of total free amino acids in soil solutions and soil extracts. Soil Biology & Biochemistry. 2002; 34(12): 1893–1902. doi: Pii s0038-0717(02)00203-1 10.1016/s0038-0717(02)00203-1 PMID: 000183851300008.

[9] Gioseffi E, de Neergaard A, Schjoerring JK. Interactions between uptake of amino acids and inorganic nitrogen in wheat plants. Biogeosciences. 2012; 9(4): 1509–1518. 10.5194/bg-9-1509-2012 PMID: 00304049800017.

[10] Öhlund J, Näsholm T. Regulation of organic and inorganic nitrogen uptake in Scots pine (*Pinus sylvestris*) seedlings. Tree Physiology. 2004; 24(12): 1397–1402. PMID: 000225591200009. 15465702 10.1093/treephys/24.12.1397

[11] Wang XL, Ye J, Gonzalez Perez P, Tang DM, Huang DF. The impact of organic farming on the soluble organic nitrogen pool in horticultural soil under open field and greenhouse conditions: a case study. Soil Science and Plant Nutrition. 2013; 59(2): 237–248. 10.1080/00380768.2013.770722 PMID: 000319325300013.

[12] Jämtgård S, Näsholm T, Huss-Danell K. Nitrogen compounds in soil solutions of agricultural land. Soil Biology and Biochemistry. 2010; 42(12): 2325–2330. 10.1016/j.soilbio.2010.09.011

[13] Vidal EA, Moyano TC, Riveras E, Contreras-Lopez O, Gutierrez RA. Systems approaches map regulatory networks downstream of the auxin receptor AFB3 in the nitrate response of *Arabidopsis thaliana* roots. Proceedings of the National Academy of Sciences of the United States of America. 2013; 110(31): 12840–12845. 10.1073/pnas.1310937110 PMID: 000322441500075. 23847199 PMC3732920

[14] Forde BG. Nitrogen signalling pathways shaping root system architecture: An update. Current Opinion in Plant Biology. 2014; 21: 30–36. 10.1016/j.pbi.2014.06.004 PMID: 000345255300006. 24997289

[15] Forde BG. Glutamate signalling in roots. Journal of Experimental Botany. 2014; 65(3): 779–787. 10.1093/jxb/ert335 PMID: 000331815300003. 24151303

[16] Hu L, Yu J, Liao W, Zhang G, Xie J, Lv J, et al Moderate ammonium: nitrate alleviates low light intensity stress in mini Chinese cabbage seedling by regulating root architecture and photosynthesis. Scientia Horticulturae. 2015; 186: 143–153.

[17] Zhang HM, Forde BG. An *Arabidopsis* MADS box gene that controls nutrient-induced changes in root architecture. Science. 1998; 279(5349): 407–409. 10.1126/science.279.5349.407 PMID: 000071570800053. 9430595

[18] Zhang HM, Jennings A, Barlow PW, Forde BG. Dual pathways for regulation of root branching by nitrate. Proceedings of the National Academy of Sciences of the United States of America. 1999; 96(11): 6529–6534. 10.1073/pnas.96.11.6529 PMID: 000080527100111. 10339622 PMC26916

[19] Leblanc A, Segura R, Deleu C, Le Deunff E. In low transpiring conditions, uncoupling the *BnNrt2*.*1* and *BnNrt1*.*1* NO3- transporters by glutamate treatment reveals the essential role of *BnNRT2*.*1* for nitrate uptake and the nitrate-signaling cascade during growth. Plant signaling & behavior. 2013; 8(2): e22904. 23299418 10.4161/psb.22904 PMC3656991

[20] Walch-Liu P, Forde BG. Nitrate signalling mediated by the *NRT1*.*1* nitrate transporter antagonises L-glutamate-induced changes in root architecture. Plant Journal. 2008; 54(5): 820–828. 10.1111/j.1365-313X.2008.03443.x PMID: 000256142200004. 18266918

[21] Walch-Liu P, Liu L-H, Remans T, Tester M, Forde BG. Evidence that L-glutamate can act as an exogenous signal to modulate root growth and branching in *Arabidopsis thaliana*. Plant and Cell Physiology. 2006; 47(8): 1045–1057. 10.1093/pcp/pcj075 PMID: 000240328700003. 16816406

[22] Guo Q, Love J, Roche J, Song J, Turnbull MH, Jameson PE. A RootNav analysis of morphological changes in *Brassica napus* L. roots in response to different nitrogen forms. Plant Growth Regulation. 2017; 83(1): 83–92. 10.1007/s10725-017-0285-0 PMID: 000407960700007.

[23] Kim TH, Kim EC, Kim SW, Lee HS, Choi DW. Exogenous glutamate inhibits the root growth and increases the glutamine content in *Arabidopsis thaliana*. Journal of Plant Biology. 2010; 53(1): 45–51. 10.1007/s12374-009-9084-0 PMID: 000276075100006.

[24] Domínguez-May AV, Carrillo-Pech M, Barredo-Pool FA, Martínez-Estévez M, Us-Camas RY, Moreno-Valenzuele OA, et al A novel effect for glycine on root system growth of habanero pepper. Journal of the American Society for Horticultural Science. 2013; 138(6): 433–442. PMID: 000333230400004.

[25] Thornton B, Osborne SM, Paterson E, Cash P. A proteomic and targeted metabolomic approach to investigate change in *Lolium perenne* roots when challenged with glycine. Journal of Experimental Botany. 2007; 58(7): 1581–1590. 10.1093/jxb/erl294 17431027

[26] Gruffman L, Jämtgärd S, Näsholm T. Plant nitrogen status and co-occurrence of organic and inorganic nitrogen sources influence root uptake by Scots pine seedlings. Tree Physiology. 2014; 34(2): 205–213. 10.1093/treephys/tpt121 PMID: 000332417200010. 24488801

[27] Song S, Li G, Sun G, Liu H, Chen R. Uptake kinetics of different nitrogen forms by Chinese kale. Communications in Soil Science and Plant Analysis. 2016; 47(11): 1372–1378. 10.1080/00103624.2016.1178279 PMID: 000379551300003.

[28] Thornton B, Robinson D. Uptake and assimilation of nitrogen from solutions containing multiple N sources. Plant Cell and Environment. 2005; 28(6): 813–821. 10.1111/j.1365-3040.2005.01332.x PMID: 000229803800010.

[29] Zheng D, Han X, An Y, Guo H, Xia X, Yin W. The nitrate transporter *NRT2*.*1* functions in the ethylene response to nitrate deficiency in *Arabidopsis*. Plant Cell and Environment. 2013; 36(7): 1328–1337. 10.1111/pce.12062 PMID: 000319875100008. 23305042

[30] Iqbal N, Khan NA, Ferrante A, Trivellini A, Francini A, Khan MIR. Ethylene role in plant growth, development and senescence: Interaction with other phytohormones. Frontiers in Plant Science. 2017; 8(9): 475 10.3389/fpls.2017.00475 28421102 PMC5378820

[31] Wang KLC, Li H, Ecker JR. Ethylene biosynthesis and signaling networks. Plant Cell. 2002; 14: S131–S151. 10.1105/tpc.001768 PMID: 000176187500010. 12045274 PMC151252

[32] Khan MIR, Trivellini A, Fatma M, Masood A, Francini A, Iqbal N, et al Role of ethylene in responses of plants to nitrogen availability. Frontiers in Plant Science. 2015; 6: 927. doi: 92710.3389/fpls.2015.00927. PMID: 000364577000001. 10.3389/fpls.2015.00927 26579172 PMC4626634

[33] Tian QY, Sun P, Zhang WH. Ethylene is involved in nitrate-dependent root growth and branching in *Arabidopsis thaliana*. New Phytologist. 2009; 184(4): 918–931. 10.1111/j.1469-8137.2009.03004.x PMID: 000271644500017. 19732351

[34] Li GJ, Li BH, Dong GQ, Feng XY, Kronzucker HJ, Shi WM. Ammonium-induced shoot ethylene production is associated with the inhibition of lateral root formation in *Arabidopsis*. Journal of Experimental Botany. 2013; 64(5): 1413–1425. 10.1093/jxb/ert019 PMID: 000316271700023. 23382554

[35] Leblanc A, Renault H, Lecourt J, Etienne P, Deleu C, Le Deunff E. Elongation changes of exploratory and root hair systems induced by aminocyclopropane carboxylic acid and aminoethoxyvinylglycine affect nitrate uptake and *BnNrt2*.*1* and *BnNrt1*.*1* transporter gene expression in oilseed rape. Plant Physiology. 2008; 146(4): 1928–1940. 10.1104/pp.107.109363 PMID: 000256417900036. 18287493 PMC2287360

[36] Luo JK, Sun SB, Jia LJ, Chen W, Shen QR. The mechanism of nitrate accumulation in pak choi *Brassica campestris* L.*ssp Chinensis* (L.). Plant and Soil. 2006; 282(1–2): 291–300. 10.1007/s11104-005-6094-7 PMID: 000238501700023.

[37] Stoelken G, Simon J, Ehlting B, Rennenberg H. The presence of amino acids affects inorganic N uptake in non-mycorrhizal seedlings of European beech (*Fagus sylvatica*). Tree Physiology. 2010; 30(9): 1118–1128. 10.1093/treephys/tpq050 PMID: 000280923800007. 20595637

[38] Islam E, Yang X, Li T, Liu D, Jin X, Meng F. Effect of Pb toxicity on root morphology, physiology and ultrastructure in the two ecotypes of *Elsholtzia argyi*. Journal of Hazardous Materials. 2007; 147(3): 806–816. 10.1016/j.jhazmat.2007.01.117 PMID: 000249262600016. 17343984

[39] Yu Y, Jin C, Sun C, Wang J, Ye Y, Zhou W, et al Inhibition of ethylene production by putrescine alleviates aluminium-induced root inhibition in wheat plants. Scientific Reports. 2016; 6: 18888. doi: 1888810.1038/srep18888. PMID: 000368678000001. 10.1038/srep18888 26744061 PMC4705537

[40] Liu XQ, Ko KY, Kim SH, Lee KS. Enhancement of nitrate uptake and reduction by treatment with mixed amino acids in red pepper (*Capsicum annuum* L.). Acta Agriculturae Scandinavica, Section B—Soil & Plant Science. 2007; 57(2): 167–172. 10.1080/09064710600914277

[41] Wang HJ, Wu LH, Wang MY, Zhu YH, Tao QN, Zhang FS. Effects of amino acids replacing nitrate on growth, nitrate accumulation, and macroelement concentrations in pak choi (*Brassica chinensis* L.). Pedosphere. 2007; 17(5): 595–600. 10.1016/s1002-0160(07)60070-8 PMID: 000249943600006.

[42] Tang YF, Sun XC, Hu CX, Tan QL, Zhao XH. Genotypic differences in nitrate uptake, translocation and assimilation of two Chinese cabbage cultivars [*Brassica campestris* L. *ssp Chinensis* (L.)]. Plant Physiology and Biochemistry. 2013; 70: 14–20. 10.1016/j.plaphy.2013.04.027 PMID: 000323355700003. 23770590

[43] Bai L, Deng H, Zhang X, Yu X, Li Y. Gibberellin is involved in inhibition of cucumber growth and nitrogen uptake at suboptimal root-zone temperatures. PLoS ONE. 2016; 11(5): e0156188. doi: e015618810.1371/journal.pone.0156188. PMID: 000376880200068. 10.1371/journal.pone.0156188 27213554 PMC4877016

[44] Princi MP, Lupini A, Longo C, Miller AJ, Sunseri F, Abenavoli MR. Long- and short-term effects of boron excess to root form and function in two tomato genotypes. Plant Physiology and Biochemistry. 2016; 109: 9–19. 10.1016/j.plaphy.2016.08.023 PMID: 000389389700002. 27620270

[45] Soper FM, Paungfoo-Lonhienne C, Brackin R, Rentsch D, Schmidt S, Robinson N. *Arabidopsis* and *Lobelia anceps* access small peptides as a nitrogen source for growth. Functional Plant Biology. 2011; 38(10): 788–796. 10.1071/FP11077 32480936

[46] Bonner CA, Williams DS, Aldrich HC, Jensen RA. Antagonism by L-glutamine of toxicity and growth inhibition caused by other amino acids in suspension cultures of Nicotiana silvestris. Plant Science. 1996; 113(1): 43–58.

[47] Gifford ML, Dean A, Gutierrez RA, Coruzzi GM, Birnbaum KD. Cell-specific nitrogen responses mediate developmental plasticity. Proceedings of the National Academy of Sciences of the United States of America. 2008; 105(2): 803–808. 10.1073/pnas.0709559105 18180456 PMC2206617

[48] Cao X, Chen X, Li X, Yuan L, Wu L, Zhu Y. Rice uptake of soil adsorbed amino acids under sterilized environment. Soil Biology & Biochemistry. 2013; 62: 13–21. 10.1016/j.soilbio.2013.02.018 PMID: 000320425800003.

[49] Vidmar JJ, Zhuo D, Siddiqi MY, Schjoerring JK, Touraine B, Glass ADM. Regulation of high-affinity nitrate transporter genes and high-affinity nitrate influx by nitrogen pools in roots of barley. Plant Physiology. 2000; 123(1): 307–318. 10.1104/pp.123.1.307 PMID: 000087046500028. 10806247 PMC59004

[50] Robinson D. Integrated root responses to variations in nutrient supply In: BassiriRad H, editor. Nutrient acquisition by plants: An ecological perspective. Berlin, Heidelberg: Springer-Verlag 2005 pp. 43–61.

[51] Zheng SL, Cheng H, Li PH, Yuan JC. Root vigor and kinetic characteristics and nitrogen use efficiencies of different potato (*Solanum tuberosum* L.) cultivars. Journal of Agricultural Science and Technology. 2016; 18(2): 399–410. PMID: 000372063700010.

[52] Ge T, Song S, Roberts P, Jones DL, Huang D, Iwasaki K. Amino acids as a nitrogen source for tomato seedlings: The use of dual-labeled (^13^C, ^15^N) glycine to test for direct uptake by tomato seedlings. Environmental and Experimental Botany. 2009; 66(3): 357–361. 10.1016/j.envexpbot.2009.05.004

[53] Cambui CA, Svennerstam H, Gruffman L, Nordin A, Ganeteg U, Näsholm T. Patterns of plant biomass partitioning depend on nitrogen source. PLoS ONE. 2011; 6(4): e19211 10.1371/journal.pone.0019211 21544211 PMC3081341

[54] Krouk G, Mirowski P, LeCun Y, Shasha DE, Coruzzi GM. Predictive network modeling of the high-resolution dynamic plant transcriptome in response to nitrate. Genome Biology. 2010; 11(12): R123 10.1186/gb-2010-11-12-r123 21182762 PMC3046483

[55] Saiz-Fernandez N, De Diego N, Sampedro MC, Mena-Petite A, Ortiz-Barredo A, Lacuesta M. High nitrate supply reduces growth in maize, from cell to whole plant. Journal of Plant Physiology. 2015; 173: 120–129. 10.1016/j.jplph.2014.06.018 PMID: 000345633700015. 25462086

[56] Iqbal N, Nazar R, Syeed S, Masood A, Khan NA. Exogenously-sourced ethylene increases stomatal conductance, photosynthesis, and growth under optimal and deficient nitrogen fertilization in mustard. Journal of Experimental Botany. 2011; 62(14): 4955–4963. 10.1093/jxb/err204 PMID: 000295983800020. 21705383 PMC3193011

[57] De Gernier H, De Pessemier J, Xu J, Cristescu SM, Van Der Straeten D, Verbruggen N, et al A comparative study of ethylene emanation upon nitrogen deficiency in natural accessions of *Arabidopsis thaliana*. Frontiers in Plant Science. 2016; 7: 70. doi: 7010.3389/fpls.2016.00070. PMID: 000369804000001. 10.3389/fpls.2016.00070 26904047 PMC4748056

[58] Iqbal N, Umar S, Khan NA. Nitrogen availability regulates proline and ethylene production and alleviates salinity stress in mustard (*Brassica juncea*). Journal of Plant Physiology. 2015; 178: 84–91. 10.1016/j.jplph.2015.02.006 PMID: 000353783300011. 25800225

[59] Kaack K, Pedersen HL. Effects of potassium, phosphorus and nitrogen fertilization on endogenous ethylene and quality characteristics of apples (*Malus domestica* L.). Journal of Plant Nutrition. 2014; 37(7): 1148–1155. 10.1080/01904167.2013.868484 PMID: 000335212600015.

[60] Iqbal N, Trivellini A, Masood A, Ferrante A, Khan NA. Current understanding on ethylene signaling in plants: The influence of nutrient availability. Plant Physiology and Biochemistry. 2013; 73: 128–138. 10.1016/j.plaphy.2013.09.011 PMID: 000329007000017. 24095919

[61] Hu Y, Vandenbussche F, Van der Straeten D. Regulation of seedling growth by ethylene and the ethylene-auxin crosstalk. Planta. 2017; 245(3): 467–489. 10.1007/s00425-017-2651-6 PMID: 000394436100001. 28188422

[62] Caba JM, Recalde L, Ligero F. Nitrate-induced ethylene biosynthesis and the control of nodulation in alfalfa. Plant Cell and Environment. 1998; 21(1): 87–93. 10.1046/j.1365-3040.1998.00242.x PMID: 000072505000010.

