# Supplementary figures and images for: Exogenous glycine inhibits root elongation and reduces nitrate-N uptake in pak choi (Brassica campestris ssp. Chinensis L.)
Source: PLoS One. 2018 Sep 21;13(9):e0204488. doi: 10.1371/journal.pone.0204488 (PMC6150514; doi:10.1371/journal.pone.0204488)

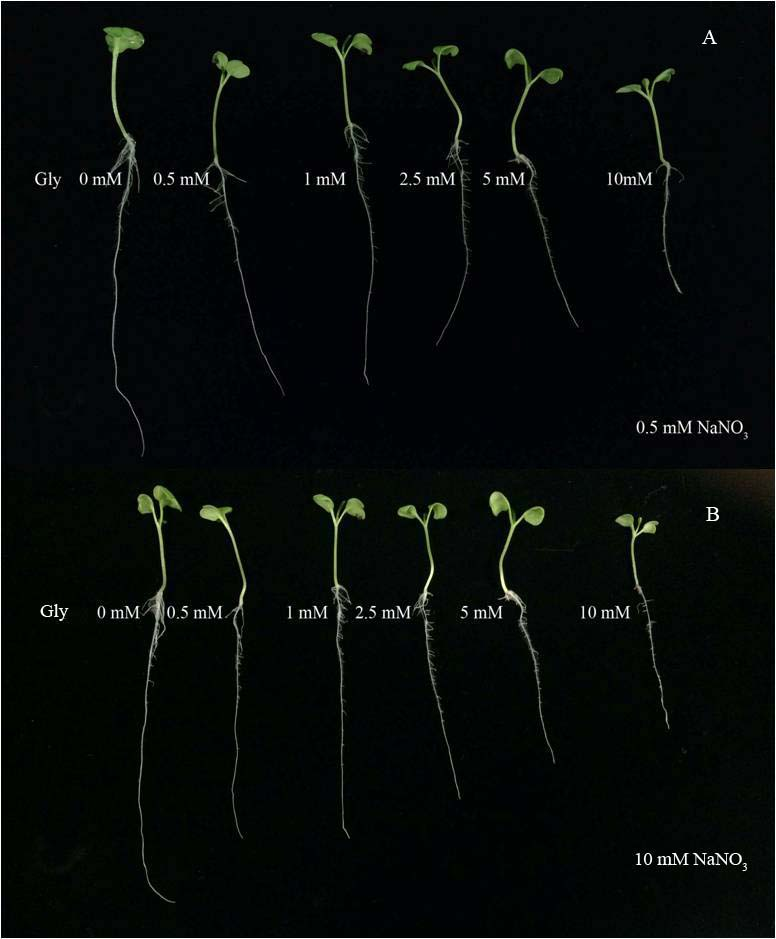

Supplement: S1 Fig — Root growth of pak choi seedlings grown on axenic agar medium containing (A) 0.5 mM or (B) 10 mM NO3--N and a range of concentrations of Gly for 5 d. (TIF) [file pone.0204488.s001.tif]

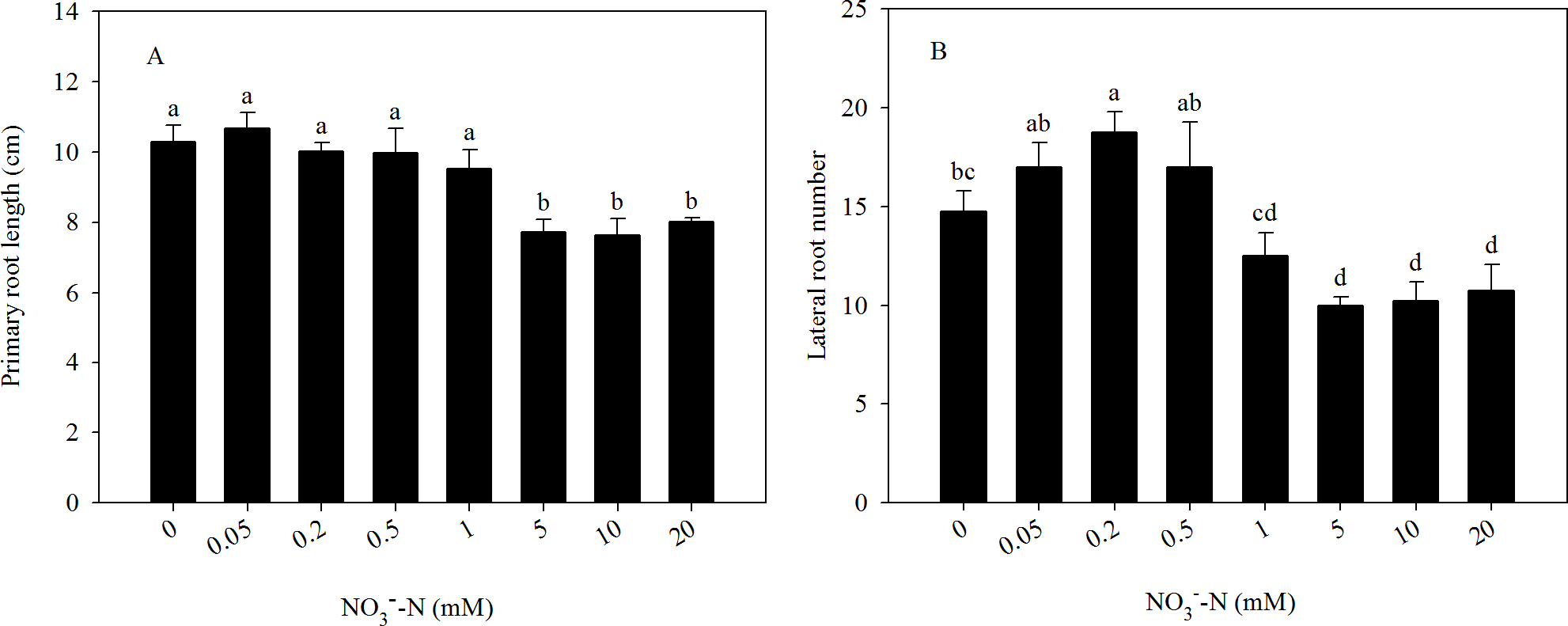

Supplement: S2 Fig — Effect of NO3--N supply on the (A) primary root length and (B) lateral root number of pak choi seedlings cultured for 4 d on agar plates. Data are mean ± SE (n = 5). Different letters indicate significant differences at P < 0.05, LSD test. (TIF) [file pone.0204488.s002.tif]

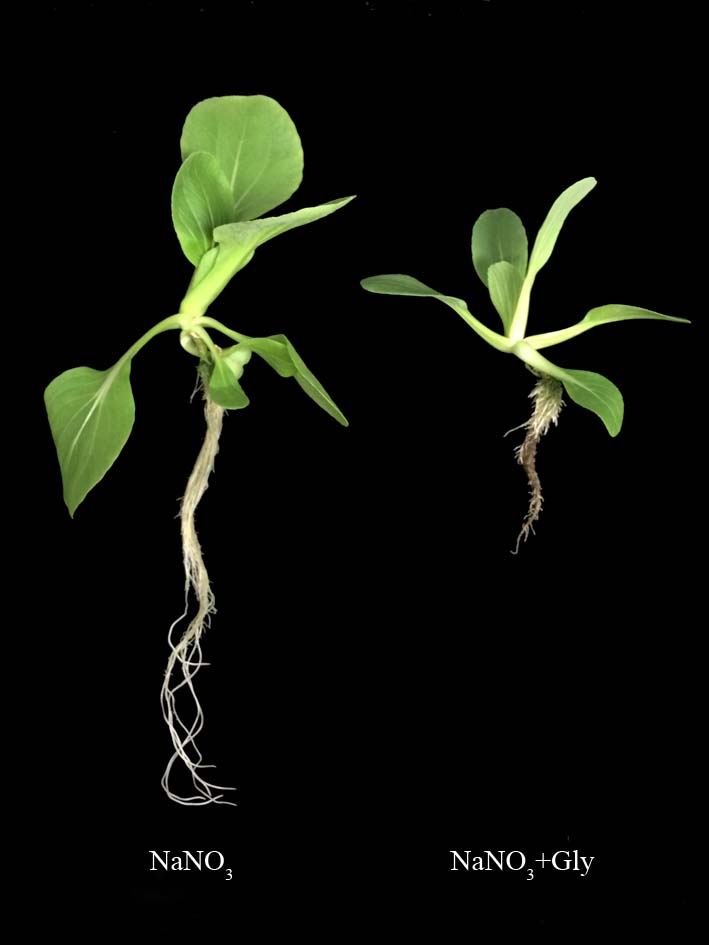

Supplement: S3 Fig — Eighteen-day-old pak choi seedlings were transferred to nutrient solution containing 10 mM NaNO3 with or without 2.5 mM Gly and harvested after 17 d. (TIF) [file pone.0204488.s003.tif]

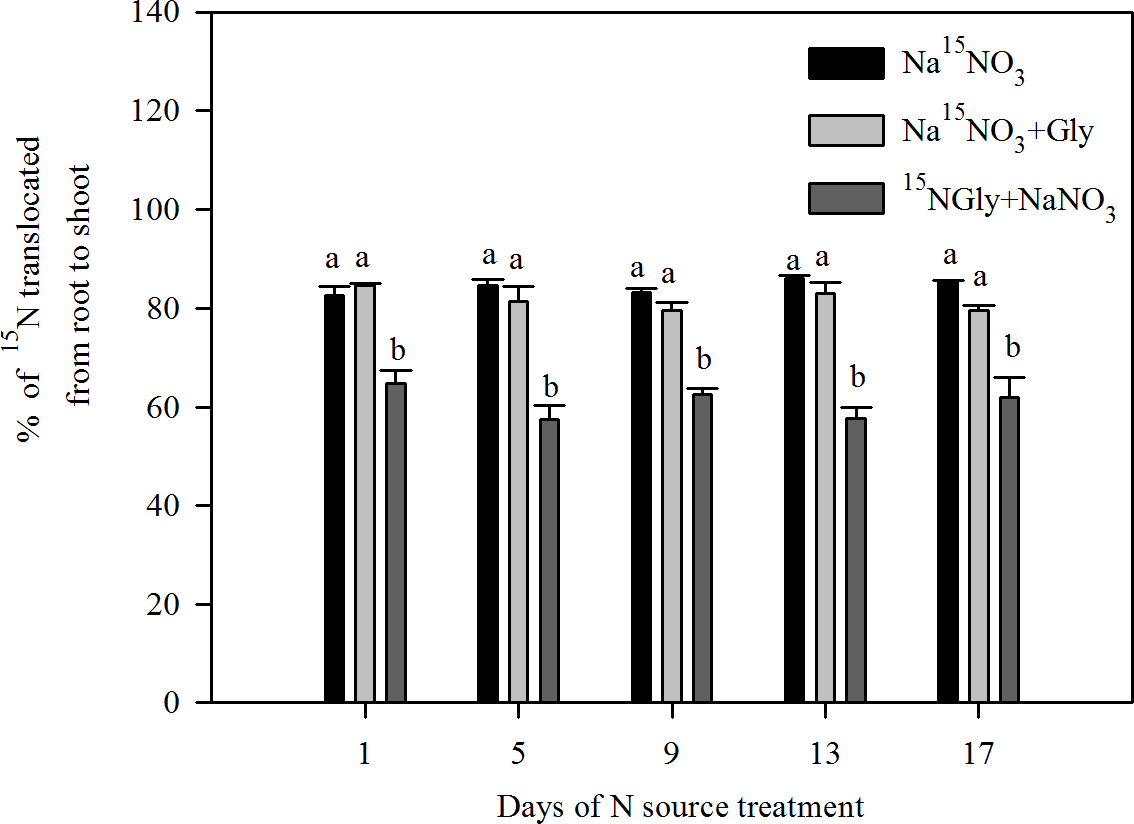

Supplement: S4 Fig — Data are mean ± SE (n = 3). Different letters indicate significant differences between treatments at P < 0.05, LSD test. (TIF) [file pone.0204488.s004.tif]

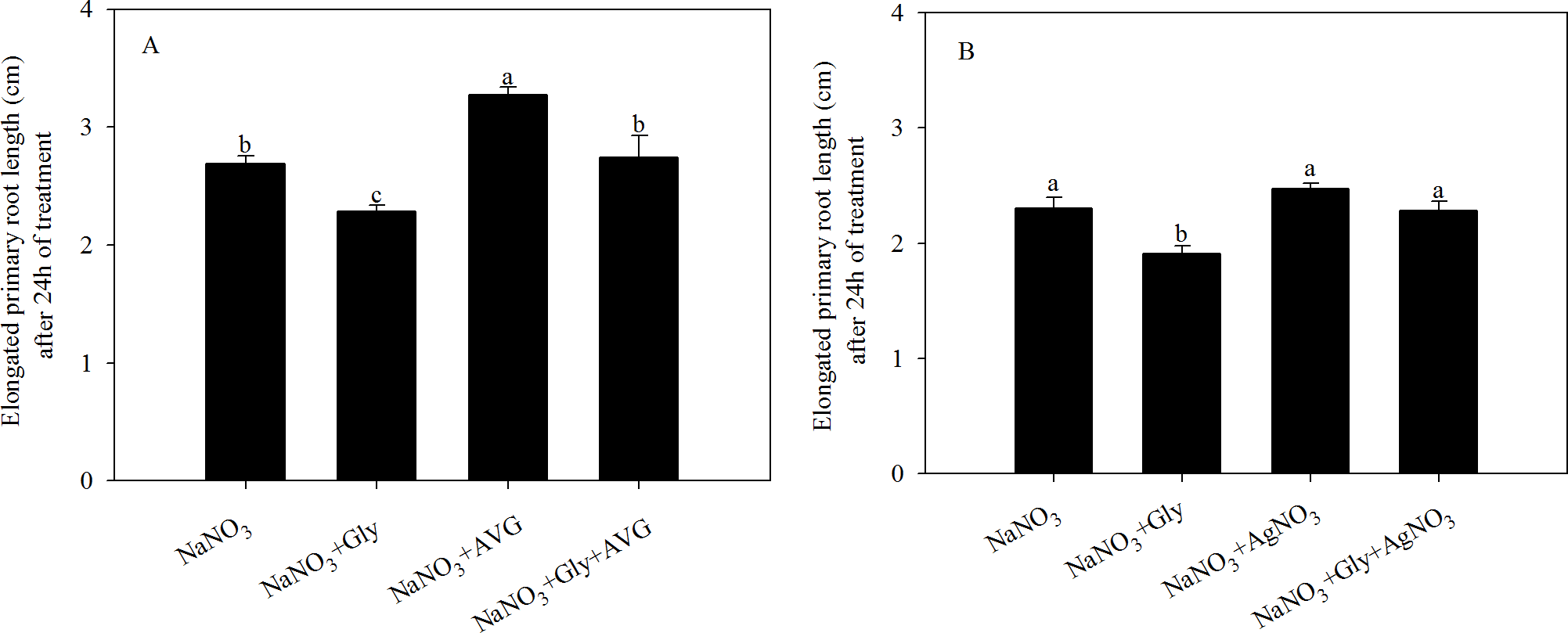

Supplement: S5 Fig — Effect of (A) 1 μM AVG and (B) 10 μM AgNO3 in the presence of 10 mM NaNO3 with or without 2.5 mM Gly on the primary root length elongation of pak choi seedlings on agar plates in the first 24 h. Data are mean ± SE (n = 5). Different letters indicate significant differences at P < 0.05, LSD test. (TIF) [file pone.0204488.s005.tif]

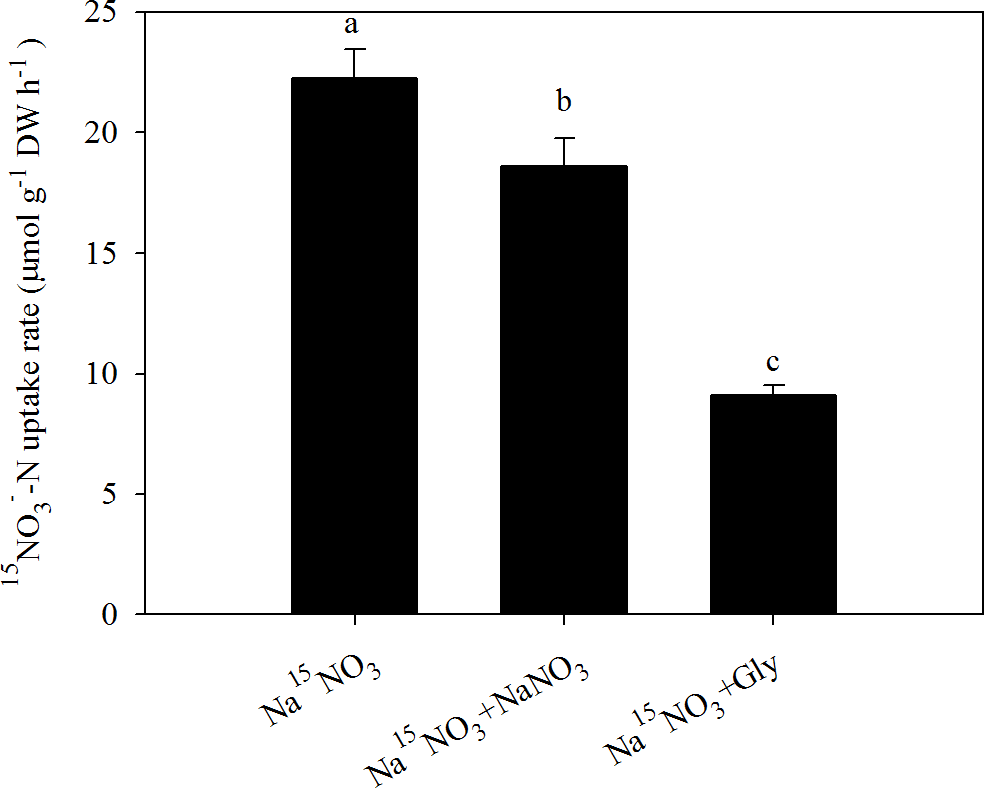

Supplement: S6 Fig — Twenty two-day-old pak choi seedlings were exposed to nutrient solution containing 10 mM Na15NO3, 10 mM Na15NO3 + 2.5 mM NO3--N, or 10 mM Na15NO3 + 2.5 mM Gly for 4 h. Data are mean ± SE (n = 3). Different letters indicate significant differences at P < 0.05, LSD test. (TIF) [file pone.0204488.s006.tif]

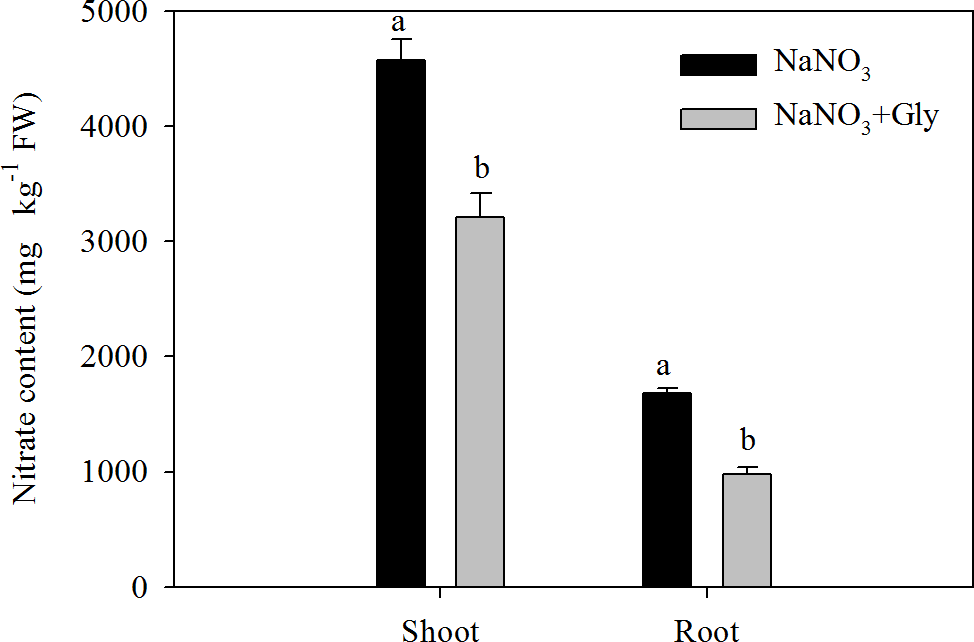

Supplement: S7 Fig — Eighteen-day-old pak choi seedlings were transferred to nutrient solution containing 10 mM NO3--N with or without 2.5 mM Gly for 5 d. Data are mean ± SE (n = 4). Different letters indicate significant differences between treatments at P < 0.05, Student’s t-test. (TIF) [file pone.0204488.s007.tif]
