# Supplementary material for: Exogenous glycine inhibits root elongation and reduces nitrate-N uptake in pak choi (Brassica campestris ssp. Chinensis L.)
Source: PLoS One. 2018 Sep 21;13(9):e0204488. doi: 10.1371/journal.pone.0204488 (PMC6150514; doi:10.1371/journal.pone.0204488)
Supplement: S1 Table — (DOT) [file pone.0204488.s008.dot]

**S1 Table. Effects of exogenous Gly on the concentrations of amino acids (μg·g^-1^ FW) in the roots of pak choi seedlings after 5 d treatment under hydroponic culture conditions**

| Treatment | Serine | Glycine | Methionine |
| --- | --- | --- | --- |
| NaNO_3_ | 29.50±0.78b | 9.79±0.13b | 3.99±0.17b |
| NaNO_3_+Gly | 496.24±19.13a | 306.30±8.12a | 5.25±0.15a |

Data are mean ± SE (*n* = 4). Different letters indicate significant differences between treatments at *P* < 0.05, Student’s *t* test.
